# Socio-demographic factors associated with self-protecting behavior during the Covid-19 pandemic

**DOI:** 10.1007/s00148-020-00818-x

**Published:** 2021-01-14

**Authors:** Nicholas W. Papageorge, Matthew V. Zahn, Michèle Belot, Eline van den Broek-Altenburg, Syngjoo Choi, Julian C. Jamison, Egon Tripodi

**Affiliations:** 1 Department of Economics, Johns Hopkins University, IZA and NBER, Balitmore, MD USA; 2 Department of Economics, Johns Hopkins University, Baltimore, MD USA; 3 Department of Economics, Cornell University and IZA, Ithaca, NY USA; 4 Larner College of Medicine, University of Vermont, Burlington, VT USA; 5 Department of Economics, Seoul National University, Seoul, Gwanak-gu South Korea; 6 University of Exeter Business School, Exeter, Devon UK; 7 Department of Economics, University of Essex, Colchester, Essex UK

**Keywords:** Covid-19, Income inequality, Social distancing, Housing, Work arrangements, J0, H0, I1

## Abstract

Given the role of human behavior in the spread of disease, it is vital to understand what drives people to engage in or refrain from health-related behaviors during a pandemic. This paper examines factors associated with the adoption of self-protective health behaviors, such as social distancing and mask wearing, at the start of the Covid-19 pandemic in the USA. These behaviors not only reduce an individual’s own risk of infection but also limit the spread of disease to others. Despite these dual benefits, universal adoption of these behaviors is not assured. We focus on the role of socioeconomic differences in explaining behavior, relying on data collected in April 2020 during the early stages of the Covid-19 pandemic. The data include information on income, gender and race along with unique variables relevant to the current pandemic, such as work arrangements and housing quality. We find that higher income is associated with larger changes in self-protective behaviors. These gradients are partially explained by the fact that people with less income are more likely to report circumstances that make adopting self-protective behaviors more difficult, such as an inability to tele-work. Both in the USA and elsewhere, policies that assume universal compliance with self-protective measures—or that otherwise do not account for socioeconomic differences in the costs of doing so—are unlikely to be effective or sustainable.

## Introduction

The spread of illness is largely influenced by human behavior. In the presence of strong externalities, a concern is that individual behavior may not align with socially optimal outcomes (Posner and Tomas J. P. 1993). This is especially salient in contexts where the costs of protective behaviors, i.e., behaviors that limit the spread of illness, are unevenly distributed across socio-demographic groups (Pampel et al. 2010). For instance, in the Covid-19 pandemic, individuals who face a relatively low risk of serious illness, but who are economically vulnerable (e.g., lacking comfortable housing, the ability to work from home) may not follow recommendations or directives to engage in protective behaviors, such as wearing a mask or social distancing. This potentially puts high-risk groups in danger of infection and prolongs the pandemic.

The socially optimal amount of protective behaviors—the levels that balance public health concerns with individual burdens and aggregate economic costs—are not yet fully understood, and are unlikely to be for some time due to uncertainty about the virus and about which behaviors most effectively prevent its spread (Manski 2020). Compounding this uncertainty, we do not yet understand the full extent of the economic and social costs of the pandemic, ranging from job losses, shuttered businesses, and gaps in schooling, to violence and addiction, among others (Fairlie et al. 2020; Alon et al. 2020; Mongey et al. 2020; Coibion et al. 2020; Viner et al. 2020). Nevertheless, understanding what factors affect individuals’ incentives to engage in protective behaviors will be of critical importance as we develop effective and humane policy, evaluate the current epidemic, make plans to emerge from it, and begin to prepare for future pandemics.

This paper examines factors predicting adoption of individual self-protecting behaviors at the start of the Covid-19 pandemic in the USA. We focus on the USA since the country lacks a consistent national policy on Covid-related behaviors, leading to a variety of different state-level policy responses and individual-level behavior changes (Adolph et al. 2020; Béland et al. 2020). Moreover, there is variation in local infection rates at a given point in time (Allcott et al. 2020b; Manski and Molinari 2020) along with vast socioeconomic inequality across US citizens. Together, these features of the US context allow us to examine how individuals with different characteristics and facing different policy environments respond to a pandemic. Moreover, the lessons we learn extend beyond the USA. For example, economic inequality—and, potentially, resulting variation in the costs of engaging in self-protective behaviors during an infectious disease pandemic—is present in other countries.^1^ Indeed, non-compliance with self-protective measures is not unique to the USA. Starting in the Fall of 2020, Europe has experienced a “second wave” of the Covid-19 pandemic, driven in part by people opting to not comply with local public health measures (WHO 2020).

Our study uses unique survey data collected during the third week of April 2020 and detailed in Belot et al. (2020b). The data set follows roughly 6000 individuals in 6 different countries and includes about 1000 individuals from the US, who are the focus on this study. Roughly 250 individuals come from each of four US states: California, Florida, New York and Texas. The specific factors we study include income, socio-demographic variables (e.g., race and gender), pre-pandemic health characteristics, job and income losses due to the pandemic, work arrangements (e.g., the ability to tele-work) and housing quality (e.g., access to outside space at home), along with beliefs and perceptions about the pandemic (e.g., whether individuals perceive social distancing to be an effective measure and the consequences of infection). We study how these factors relate to three measures of behavior change from before the pandemic to the point of data collection. In particular, respondents were asked whether they changed any behavior at all in response to the pandemic; whether they increased social distancing, which includes avoiding public spaces, running fewer errands and visiting friends and family less often; and whether they increased hand washing or mask wearing. The data have information on a host of additional self-protective behaviors (and similar income gradients emerge when we examine them). We chose to focus on these three measures because they illustrate the wide range of possible self-protective behaviors: the first is very broad, including any change in behavior at all; the second is a relatively high-cost activity; and the third is a relatively low-cost activity.^2^

**Fig. 1:**
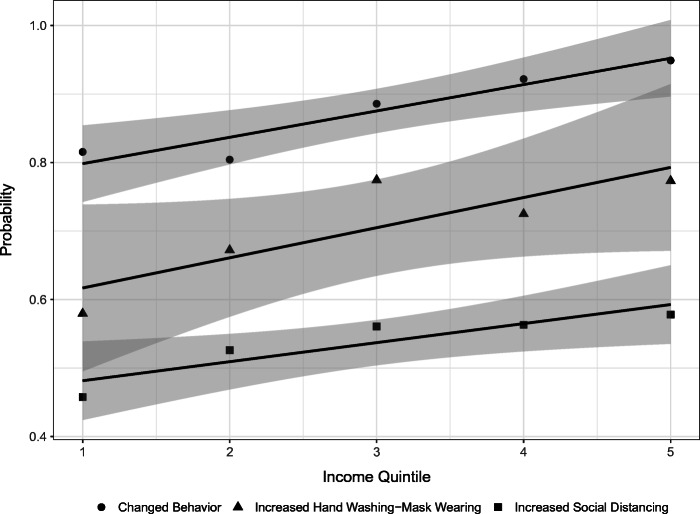
Probability of changes in self-protective behaviors by income quintile: This figure shows the proportion of US respondents within a household income quintile that reports changes in any self-protective behaviors; reports increased social distancing; and increased hand washing-mask wearing. We also plot the estimated trend relating each changed behavior to income quintiles. The gray area is the associated 95% confidence interval

We begin by documenting a striking and robust pattern apparent in the data: higher income is associated with larger increases in self-protective behaviors. Figure 1 illustrates this relationship for the three aforementioned behaviors for the US sample. Note, the figure plots changes rather than levels. Using these measures, we show that on average individuals in the fifth income quintile (quintile mean $233,895) are between 13 and 19 percentage points (16–54%) more likely to increase their self-protective behaviors compared to individuals in the first income quintile (quintile mean $13,775).^3^ For each of these behaviors, the difference between the first and fifth income quintile is statistically significant at the 1% level. It is worth noting that some of the stronger income differences in the figure appear when we compare low versus middle and high income. For example, hand washing and mask wearing changes are larger for middle versus low-income individuals, but are not larger for high- versus middle-income individuals. While increases in social distancing appear to increase in magnitude across the income distribution, we cannot reject that individuals in third, fourth and fifth quintiles increase the behavior the same amount. For changes in any behavior, we find significant rises across the income distribution. These finding suggest that higher income is generally associated with larger increases in self-protective behaviors and, moreover, that some differences are concentrated at the bottom of the income distribution, meaning that low-income individuals may face especially large costs to adopting such behaviors. Our subsequent analysis aims to shed light on these relationships by assessing which specific factors related to income help to explain differences in behavior change.

In particular, our main analysis explores these relationships between income, the pandemic and self-protective behaviors in two ways. First, we ask how income relates to initial consequences of the pandemic along with other factors that could affect social distancing and other self-protective behaviors. We illustrate the burdens lower income respondents face, ranging from increased chances of job and income losses due to the pandemic to limited access to remote work or to open air space at their residences. Taken together, this means that we would expect these people to have a harder time adopting social distancing behaviors, which may prolong the pandemic.

Next, we examine which socio-demographic characteristics predict self-protecting behaviors. To do so, we estimate a series of linear probability models where a change in self-protective behaviors is the outcome variable and the predictor variables include income along with different sets of additional variables, culminating in a final specification that includes income along with all variables. Several patterns emerge. Work arrangements and housing characteristics, particularly transitioning to tele-working and access to outside space at home, are associated with adopting more self-protecting behaviors. We also find that income losses due to the pandemic are associated with larger increases in the behaviors that we examine. Surprisingly, we find no meaningful patterns between pre-existing health conditions and increases in self-protective actions. We also show that males and respondents from Florida and Texas are less likely to engage in self-protective behaviors compared to females and respondents from California and New York, respectively. Moreover, beliefs about the effectiveness of social distancing along with perceived benefits (e.g., more time spent with family) correlate with larger increases in self-protective behaviors, though less so for lower income groups, which we discuss below. Finally, we demonstrate that the income gradient is only partially explained by the inclusion of these variables. The size of the income coefficient estimates are fairly stable across specifications where different sets of controls are included. These findings suggest that while income is an important predictor of behavior change, this relationship is only partly explained by the other factors we examine. There are likely to be other variables not captured by our data that help to further explain why income matters so much in the adoption of self-protecting behaviors.

Broadly, our findings are consistent with two key ideas. One, the initial economic consequences of the pandemic are particularly harmful to low-income individuals. Two, behaviors that stem from the pandemic could place relatively large burdens on individuals with lower incomes. For example, higher income individuals are more likely to report being able to work from home and more likely to have transitioned to tele-working instead of losing their job. As a result, adoption of self-protective behaviors, such as social distancing, are more practical, comfortable and feasible for people with more income, which is evident in the behavior changes depicted in Fig. 1. Effective and sustainable pandemic policy should be based not on what policymakers wish individuals would do, but what they expect them to do. To the extent that socioeconomic status helps to predict behavior, it should play a role in the development of policy. Further, to the extent that specific and policy-relevant factors help to explain income gradients, they could shed light on new avenues to mitigate the harm of a pandemic. For example, our results suggest the importance of outside space at home in predicting the adoption of self-protective behaviors. If so, opening parks, which may seem frivolous and unnecessarily risky at first glance, may actually be a prudent policy to prioritize. Doing so could potentially encourage self-protective behaviors by providing outside space to those who lack access to it.

The remainder of this paper proceeds as follows. In Section 2, we provide an overview of the existing literature on Covid-19 pandemic and socioeconomic health gradients. In Section 3, we provide a brief history of the Covid-19 pandemic in the USA. Section 4 discusses our data source and provides a preliminary data analysis. We then discuss the results from our main analysis in Section 5, which quantify the associations between individual characteristics and behavior changes. Section 6 concludes.

## Literature review

This paper relates to a number of ongoing research efforts that, on a nearly daily basis, provide new information on the current pandemic and people’s responses to it. To the degree our questions and findings overlap, we provide crucial replication in an era of rapid-response, hastily completed research. When questions or focus differ, we provide new information that other efforts do not. Finally, if we provide contradictory answers to similar questions, this is also important since it highlights where further research is needed.

Many studies have examined the connection between individual behaviors and the spread of disease. For example, Adda (2016) examines the spread of viral diseases stemming from economic activity. Others have looked at the role of human transmission in the Covid-19 pandemic. Qiu et al. (2020) document the role of human mobility in spreading Covid-19 in China. Their analysis highlights the importance of public health policies that limit mobility as a means of lowering transmission. Bonacini et al. (2021) explore the case of Italy, evaluating the effectiveness of different lockdown policies, showing that the government’s initial measure of closing all schools and universities was most effective at slowing the growth of Covid-19 cases in the country. The impact of human behavior on disease spread has also been documented when looking at multiple countries. For example, Milani (2021) highlights the role of social networks in the transmission of Covid-19 within and across countries. Zimmermann et al. (2020) reach similar conclusions about the impact of human behavior on the spread of the pandemic, arguing that the best strategy to mitigate future pandemics is to understand the human factor and move swiftly to mitigate it at the start of an outbreak. Our results are in line with this view. Our focus is on identifying which specific “human factors” related to socioeconomic status seem to predict behavior change and should thus be taken into account when responding to a public health crisis such as a pandemic.

Other studies have examined the economic impacts of the pandemic. For example, Adams-Prassl et al. (2020a) conduct a nationally representative survey of the UK, US and German population on the labor market impacts of Covid-19. Their findings highlight heterogeneous impacts across countries. In particular, employees in Germany were affected less by the crisis than individuals in other countries due to a well-established unemployment insurance system. Still, the pandemic exacerbated existing inequalities within each country. Another is Béland et al. (2020), which quantifies the economic impacts on wages and employment from stay at home orders in various American states. They demonstrate that despite significant economic losses from the pandemic, these orders were cost effective when taking into account the value of a statistical-life. Other studies have focused on work arrangements such as work from home. Examples include Bonacini et al. (2021), Saltiel (2020), Okubo (2020), and Rahman (2020), which examine the types of occupations and workers that have the ability to work from home and how they have been affected by the Covid-19 pandemic in countries outside the USA. A component of our contribution is linking individual demographics to access to tele-working in the USA. Beyond this descriptive exercise, we are able to link this work arrangement to income and self-protective behaviors during the pandemic. This connection helps us highlight how the burden of measures such as social distancing fall heaviest on lower income groups.

A related set of papers provides evidence of differences in the effects of the pandemic by socioeconomic status. Mongey et al. (2020) analyze the characteristics of workers in jobs that are most likely to be affected by social distancing measures. They demonstrate that these people are more economically vulnerable and live in areas that engage in less social distancing.^4^ Our paper makes a direct contribution to this line of literature. The data set we use in this paper was designed to capture detailed information about economic losses individuals experienced. Examples include work transitions, realized monetary losses and expected monetary losses. By directly linking economic impacts to individual behaviors, we are able to provide a clear picture of what factors predict observed behavior discrepancies, which in turn suggests policies that could encourage more widespread adoption of behaviors that slow the pandemic.

Indeed, a separate strand of the Covid-19 literature closely related to our study examines how individual characteristics and demographics predict or influence behaviors during the pandemic. For example, Ashraf (2020) explores the relationship between socioeconomic factors, government policy and Covid-19 health outcomes using a rich panel data set covering 80 countries. The author finds a strong negative association between Covid-19 cases and socioeconomic conditions, which can be alleviated by government policy. Other papers, such as Andersen (2020), Chiou and Tucker (2020), and Wright et al. (2020), rely on cell phone data to track individual behavior during the pandemic. These papers have documented that the adoption of social distancing measures varies across observable characteristics, such as average neighborhood income. These papers have the benefit of bypassing individual reporting, which may be biased, a point we return to when presenting results. Our paper generally corroborates findings using cell phone data. Additionally, our survey data permits a more granular analysis in that we can examine a richer set of individual-level variables, such as beliefs about social distancing and detailed work transitions. Finally, Wozniak (2020) uses a unique US data set, also publicly available, to relate disease exposure to decisions to work or take protective measures. She finds that people with Covid-19 exposure continue to work at similar rates as the non-exposed, and people with elevated risk for contracting the disease do not reduce work hours or take protective measures. We place greater attention on the associations between an individual’s protective behaviors and their characteristics and beliefs, while she focuses more on protective behaviors through the lens of risks factors (i.e., an individual’s susceptibility and potential spreading) and protective behaviors.^5^

In the US context, many papers have also examined links between political affiliation and self-protective behaviors. Examples include Adolph et al. (2020), Allcott et al. (2020a), Barrios and Hochberg (2020), Painter and Qiu (2020), and Simonov et al. (2020). In general these studies find that individuals that identify as Republicans or who live in areas with higher levels of support for President Trump and Republican elected officials are less likely to engage in self-protective behaviors or adhere to shelter in place orders. While we lack data on political affiliations, we find similar patterns when looking at individuals located in Florida and Texas, two states that voted for Trump in 2016 and 2020 and are led by governors that are vocal supporters of President Trump and his administration.

More broadly, this paper relates to a vast literature studying how socio-demographic characteristics associate with health and health behaviors. While our contribution reports evidence in a very specific context, the Covid-19 pandemic, it is noteworthy that many of the same relationships found in other health contexts are evident here.^6^ In other words, well-documented socio-demographic differences in health behaviors—and resulting health disparities—extend to the current pandemic (Yancy 2020). What we learn about behavior during a pandemic, a period when stakes are high and shifts in behavior are swift and large, can help us to understand health behavior differences more generally. As a concrete example, if we learn that certain types of work arrangements prevent social distancing, such arrangements may prevent a host of other healthy behaviors that are unrelated to the pandemic. In this way, the current pandemic can provide useful directions for future research on health behaviors and health disparities.

Finally, this paper relates to a literature examining the tension between individual behavior and public health in the presence of externalities. A key historical analogy is the HIV epidemic.^7^ In that context, reduction of risky sex behavior not only protected individuals, but also slowed the spread of the virus, which is socially beneficial. The social benefit means there is a positive externality and thus potentially a sub-optimally low level of safer sex. In the current context, the tension between private behavior and public health is exacerbated by the fact that many of the people asked to incur the most brutal economic and social costs of protective behaviors face relatively low personal risk of serious health problems. This opens up a host of broad and general ethical questions about who should bear the greatest costs to protect public health. It also casts doubt on the sustainability of policies that presume full compliance.^8^

## Covid-19 in the USA

As our focus is on the response to the Covid-19 pandemic, it is useful to recall the circumstances respondents were facing at the time our data were collected (the third week of April 2020). The first confirmed Covid-19 case in the USA was announced on January 20, 2020. About a week later, the White House Coronavirus Task Force began meeting on a daily basis. Over the next 13 days, cases in the USA remained relatively low but grew in other countries. On February 2, 2020, President Trump restricted entry into the United States by non-citizens who were physically present in China. Over the month of February, cases in the USA remained relatively low but the virus was being closely monitored by the government. On February 29, 2020, Dr. Anthony Fauci stated that Americans did not need to change their daily practices and that the current risk of the virus was low, while cautioning circumstances may change. The government maintained this message for the next week and a half. For example, on March 8, 2020, Dr. Fauci encouraged the public to not wear masks and reserve them for use by healthcare professionals and those who had fallen ill (Goodman and Schulkin 2020).

The situation took a grave turn a few days later. On March 11, 2020, the World Health Organization officially declared Covid-19 a global health pandemic. Later that day during a prime-time Oval Office address, President Trump announced additional travel restrictions to the USA from 26 European countries. Two days later, the president declared the coronavirus a national emergency. During the first two weeks of March, case counts rose steadily. By the third week of March, hospitals reported severe shortages of testing supplies, personal protective equipment and other critical supplies. Near the end of March, President Trump signed the CARES Act, a wide-ranging measure aimed at curbing the economic effects of the pandemic and procuring additional supplies to treat the disease. By this time, over 10 million Americans had filed for unemployment benefits (Goodman and Schulkin 2020).

Towards the end of April, when our survey data were collected, cumulative cases in the USA reached nearly 1 million and Covid-19 was the leading cause of death in the country. On April 23, the final day of our data collection, Mr. Trump infamously advised citizens that the digestion of bleach may be an effective way to combat the virus (Goodman and Schulkin 2020). This is an example of the depths of misinformation being shared with Americans about the pandemic and how to best guard themselves from infection. It is possible that the release and amplification of this content through certain media could explain observed income gradients for self-protective behaviors. For example, Simonov et al. (2020) explore how regional compliance with social distancing varies with Fox News viewership. To address questions about misinformation, we include beliefs about the effectiveness of social distancing in our empirical analysis.

From the start of the pandemic, the White House largely relied on state and local governments to devise and implement public health measures to combat the virus, offering virtually no unified national strategy. This pattern is highlighted by the states included in our survey sample. As case counts rose steadily during the first two weeks of March, California was the first state to take large-scale action. On March 19, Democratic Governor Gavin Newsom ordered all 40 million Californians to stay at home as much as possible, closing all non-essential businesses (Arango and Cowan 2020). The next day, New York’s Democratic Governor Andrew Cuomo issued a similar directive, noting that cases had risen from 0 to 2900 in just over 2 weeks (De Avila 2020). New York would go on to become one of the most heavily affected states by the pandemic. Florida’s Republican Governor Ron DeSantis did not implement any sort of lockdown order until April 1, 2020, when the number of cases surpassed 7000. In fact, the day before the measure was announced, Governor DeSantis said he had no plans to issue any statewide measure because the White House had not instructed him to do so (Barbash and Horton 2020). Texas’ Republican Governor Greg Abbott, responded somewhat faster than Florida. Governor Abbott announced a patchwork of local restrictions on March 19, 2020 (Svitek 2020a). As cases in Texas grew, Abbott resisted calls for stronger restrictions but eventually relented, announcing a state wide stay at home order on March 31, 2020 (Svitek 2020b; 2020c).

Similar to lockdown orders, the federal government provided no unified policy on mask wearing. Trump was not observed wearing a mask publicly until July 2020 and has equivocated on when they ought to be used (Lemire 2020). Like social distancing, mask wearing policies were again deferred to states to implement as part of their own policy response. Governors Cuomo and Newsom were early proponents of masks and frequently encouraged their use before issuing mandates in April and June respectively (Higgins-Dunn et al. 2020; Romo 2020). Governor Abbott followed with a mask mandate in July (Siemaszko 2020). As of October 2020, Governor DeSantis has not mandated that masks be worn by Floridians. This summary highlights the considerable variation in the local responses to the pandemic in the states covered by our survey data.

## Data and summary statistics

### Data collection

We rely on recently collected survey data from six different countries by Belot et al. (2020b). The data were collected between April 15 and April 23 in the USA, the UK, Italy, China, Japan and Korea. The sample consists of roughly 1000 individuals in each country. As stated earlier, the focus of this paper is on the US sample, which was drawn from four states: California, Florida, New York and Texas with roughly 250 people per state. The sample is nationally representative for each country according to age, gender and household income. For the US sample, the survey is also nationally representative along race. Prior to the start of data collection, approval was obtained by the ethics board at the University of Exeter.

The survey firms Lucid and dataSpring assisted with the data collection. Respondents were initially contacted by these firms via email to participate. New invitations were sent up to the point of achieving the desired representation. Participation was remunerated according to general compensation schemes defined by the companies for their survey panelists. Respondents were prevented from taking the survey multiple times and from finishing the survey too quickly. The median response time for the survey was about 14 minutes.

Among the benefits of using this survey data set is that it contains information that is uniquely relevant to the Covid-19 pandemic. For instance, the survey collected detailed information about how a respondent’s work arrangements changed (e.g., stopped working, began tele-working). Another example is that information was collected on the characteristics of a respondent’s home, such as their current living arrangement (e.g., alone, roommates), exposure to elderly people and access to open air. This collection effort also captured information about individuals’ beliefs on several topics, ranging from the effectiveness of social distancing to how likely it is that they get infected with the virus.

While these data are valuable and informative about the Covid-19 pandemic, there are noteworthy limitations. We would prefer to use data from an ongoing study consisting of a large and representative sample, with information collected at regular intervals from the same individuals. However, to relate socioeconomic differences to Covid-19, such data simply do not exist. Thus, we must rely on survey data collected quickly. This allows us to tailor questions asked of respondents so we can answer questions about an unprecedented worldwide shock. Yet, collecting and using data in this manner has many drawbacks. First, there was a regrettable and unintentional omission: the survey did not elicit information about educational attainment.^9^ Second, while the survey is representative along important demographic characteristics including age, gender, income and race, it is not a random sample of people in the USA. As such our estimates must be interpreted with care. In particular, income groups may exhibit differences that do not extend to the whole population because there could be unobserved variables correlated to income that jointly predict survey participation and self-protection behaviors. Moreover, it is difficult to make causal claims, though this problem extends to large and representative data sets. The difference is that we lack the data that facilitate standard approaches to help to identify causal effects, such as additional variables we could use as controls or IVs or repeated observations allowing us to employ panel data methods. These shortcomings reflect the realities of conducting research rapidly and in a daily-changing environment. Still, the patterns we present here are robust and informative. We hope that the analyses and data presented in this paper can inform the development of longer term data collection efforts that aid us in better preparing for future public health crises.

### Summary statistics

Table 1 summarizes the socio-demographic characteristics and outcomes we study from the survey sample. Fifteen percent of respondents are non-white, 44% are male and about 39% are 56 years or older. Forty-five percent of respondents report at least one pre-existing health condition. While the survey data did not target pre-existing conditions for representativeness, the prevalence of pre-existing conditions in our sample closely mirrors the amount in the US population (CMS 2011; McCarthy 2020). Approximately 70% of respondents reported being employed. Among those that reported working, nearly 54% work full-time, 14% work part-time and 82% are able to work from home at the time of the survey when the pandemic was well underway. Also among these respondents, 34% report shifting to tele-work, 38% are no longer working and 20% reported no change in their employment situation. On average, respondents lost $770 in household income due to the pandemic. These were recorded as total household income losses during the first trimester of 2020.^10^

**Table 1:** Summary statistics

Variable	Value	*N*
Socio-demographics		
Non-White	0.15	1006
Male	0.44	1006
New York-California	0.54	1006
Elderly	0.39	1006
Health		
Pre-existing condition	0.45	1006
Housing		
Urban	0.41	1006
Home w/o open air access	0.15	1006
Elderly exposure	0.46	1006
Work arrangements and losses		
Working	0.70	990
Full-time | working	0.54	701
Part-time | working	0.14	701
Can work from home | working	0.82	701
Stopped working | working	0.38	701
Tele-working | working	0.34	701
Still working | working	0.20	701
Mean income quintile	3.11	1006
Mean lost HH. income ($1000)	0.77	475
Beliefs and perceptions		
Mean belief social distancing effectiveness {1–5}	3.99	1006
Mean local infection rate	0.24	971
Benefits from pandemic	0.84	1006
Outcomes		
Changed behavior	0.88	1006
Increased SD	0.54	824
Increased hand washing-mask wearing	0.71	933
*N*		1006

Summary statistics for characteristics of individuals for the entire US sample. The first column lists the probability that a randomly drawn individual has the listed characteristic or the mean value of the variable from the sample. The exceptions are full-time, part-time, work from home, stopped working, tele-working, and still working, which were computed among the sample of respondents that were observed working. The second column lists the total number of observations for which that variable had a non-missing value

The survey contains two variables about labor status. The first asks about the current work arrangement and the second asks about changes due to the pandemic. Using these two variables, we construct a single measure that captures possible ways that the pandemic has affected individuals with different work arrangements. There are five possibilities: (i) “Never Worked” refers to individuals who were not working prior to or during the pandemic (e.g., retirees); (ii) “Stopped Working” refers to people who were working (full-time, part-time or self-employed) and stopped working due to the pandemic; (iii) “Began Tele-Working” refers to people who were employed prior to the pandemic and are still employed, but transitioned to working from home due to the pandemic; (iv) “Still Working” describes individuals who were working prior to the pandemic and whose work status has not changed; and (v) “Other” includes people who report working prior to the pandemic and also report “other” when asked how their work status has changed. We should note that 17 individuals do not fit into any of the categories above due to contradictory answers. As an example, some respondents report not working before the pandemic and also transitioning to tele-working, which is difficult to categorize.^11^

Figure 5 in the Appendix summarizes this work status variable by age group and income quintile. As expected, we find that younger respondents tend to be more likely to begin tele-working whereas older workers are more likely to not be working before or during the pandemic. We also see that higher income people are more likely to transition to tele-working, while lower income people are more likely to either not be working before the pandemic or to have stopped working due to the pandemic. This pattern is telling, as tele-working likely lowers the cost of many of these self-protective behaviors, such as social distancing. The share of respondents that were still working without any change was relatively stable across income quintiles. A deeper look into the survey finds an intuitive pattern. Those that were in lower income quintiles and reported still working belong to professions such as cashiers, packers and packagers, among others. Those in higher quintiles that reported still working included lawyers, computer programmers and managers. These professions differ substantially in what they pay, but include people who have been deemed “essential workers.” This explains the lack of an income gradient for this particular category.

The survey also includes information on beliefs and perceptions. Approximately 73% believe that social distancing is either very effective or extremely effective, versus 24% who believe it is slightly or moderately effective. Only 3% of the sample believed social distancing was not effective at all. Almost 39% of respondents believed it to be extremely effective. On average, respondents believe that 24% of the people in their area are infected. This high number is driven by a mass of respondents reporting implausibly high numbers (including some saying over 90%), which we discuss below.^12^ We speculate that some of these extreme beliefs could be driven by the spread of misinformation, which has been shown to have an impact on the adoption of self-protective behaviors (Simonov et al. 2020). Finally, about 84% perceive some benefits from the pandemic (e.g., getting to spend more time with family or reducing pollution). As we show below, these perceptions predict behavior.

**Fig. 2:**
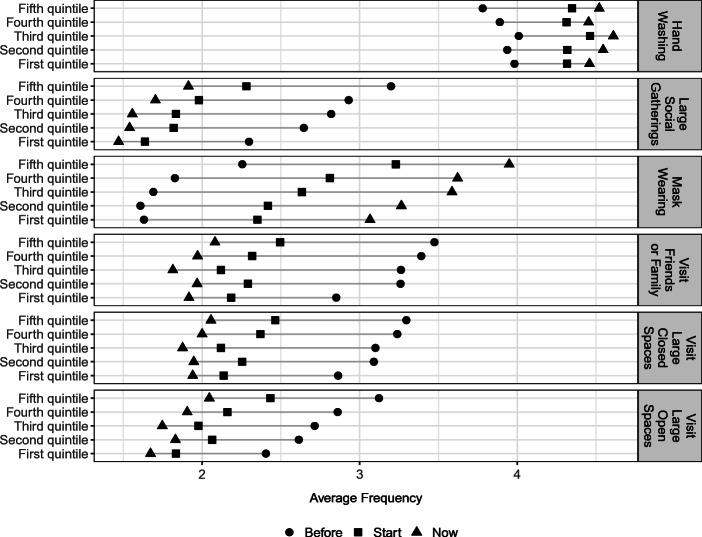
Average frequency of select behaviors by income quintile and time period: These tabulations are the average frequency respondents within an income group reported doing each listed activity. Possible responses were 1 (never), 2 (rarely), 3 (sometimes), 4 (very often), and 5 (always). These are calculated for the time period before the pandemic, at the start of the pandemic and a few weeks into the pandemic

**Fig. 3:**
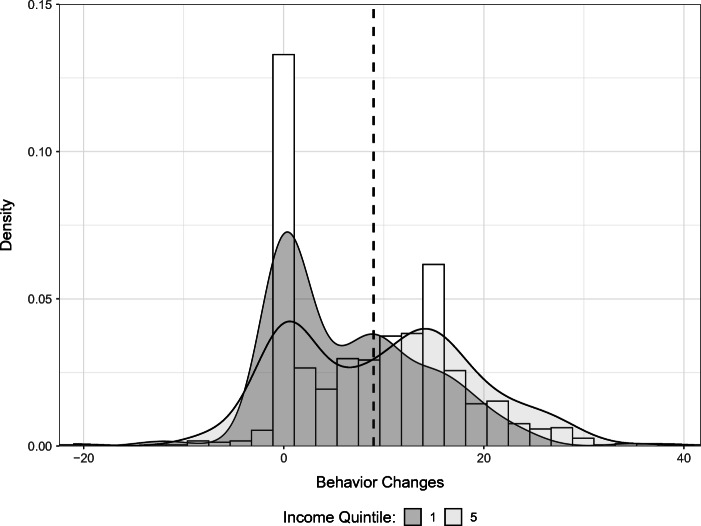
Distribution of behavior changes: Distribution of behavior changes from before the pandemic to a few weeks after the pandemic started for all 15 behaviors included in our survey data. Changes are normalized such that self-protecting changes are positive and reductions in these behaviors are negative. The densities for the lowest and highest income quintiles are broken out separately. The dashed line represents the mean number of behavior changes across all income quintiles

The survey measures behavior change in two ways. First, respondents are asked directly if they engaged in any self-protective behavior in response to the pandemic. About 88% report having done so. Second, the survey asks respondents to report how frequently they engaged in 15 different activities before the pandemic, at the start of the pandemic and a few weeks after the pandemic began. These behaviors ranged from hand washing and eating healthily to visiting large open or closed spaces and visiting friends and family. Given this data structure, we observe how the respondents changed their behaviors over time. For each behavior, respondents could answer (1) never, (2) rarely, (3) sometimes, (4) very often or (5) always. Figure 2 summarizes the average frequency with which respondents engaged in some of these behaviors at each time period.^13^ Consistent with Fig. 3, we see an increase in the average frequency of self-protective behaviors from before the pandemic to weeks after the pandemic started. To get a sense of behavior change, we construct a count variable for each individual for the number of changes towards (or away from) self-protection from before the pandemic to a few weeks after it had begun, when the data were collected. Figure 3 plots the distribution of the resulting variable for all 15 behaviors captured by our survey data. The changes are normalized such that self-protecting changes are positive, while reducing such behaviors is recorded as negative.^14^ The median number of changes towards self-protection is 9.

We highlight two main takeaways from Fig. 3. First, few people exhibit a net decline in self-protective behaviors. Second, most people either do not change their behavior much at all (note the spike in the distribution at zero) or make a fairly large number of changes. Figure 3 also presents the densities for the lowest and highest income quintiles. We find that a greater mass of low-income people are not increasing their self-protective behaviors. We see the opposite pattern for high-income people, with a greater share of high-income people adopting more self-protective behaviors. This pattern is generally consistent for the income quintiles between these two groups. For example, if we were to plot the distribution for the third quintile, its peaks would be nested between the peaks for the first and fifth income quintiles. A goal of this paper is to understand what factors explain these different behavioral responses.

In our subsequent analysis, we focus on three measures of behavior change. The first is from the aforementioned question asking respondents directly if they changed their behavior in response to the pandemic. The second is a composite behavior variable for social distancing. We focus on: visiting large open spaces, visiting large closed spaces, attending large social gatherings, and visiting with friends or family. As we are mainly interested in how behaviors change, we look at how this social distancing composite changes over time. An increase in social distancing is defined as an above-median increase in the number of self-protecting improvements for the four behaviors of interest. The third measure focuses on changes in hand washing and wearing a mask. Each of these measures provides insight into how individual behaviors are changing during the pandemic and cover a range of costs. Changing any behavior and increasing hand washing or masks wearing are relatively low-cost shifts. Increasing social distancing behaviors is more costly and may be harder for some groups of people to do.

We make four types of drops from the full US survey sample for our analyses. First, we drop respondents who did not report an income quintile or gender (15 observations). Second, as discussed previously, our composite behavior dependent variables examine increases in behaviors. We drop respondents who reported always engaging in these behaviors and who thus had no ability to increase further (40 observations for social distancing and 73 for hand washing-mask wearing). The third set of drops are the outliers in local infection rate beliefs (36 observations). Finally, we drop 17 respondents whose work status is unclear. For each dependent variable, we hold the analysis sample constant across specifications to facilitate comparisons. Thus for each outcome, we take the maximum number of observations that remain after these sets of drops.

While we are looking at changes in behaviors, it is important to note that we observe differences in the levels of engagement in these behaviors across income quintiles before the pandemic. For example, in Fig. 2 we can see that people in the fifth income quintile reported attending large social gatherings at a higher rate than those in the lowest income quintile. The level of other pre-Covid behaviors, such as hand washing and mask wearing, are more similar across income quintiles. This pattern suggests that higher income individuals may necessarily need to adopt more self-protective behaviors than lower income people, who only need to make relatively minor adjustments after the outbreak of the pandemic.^15^ Despite these larger behavior changes, higher income people may be engaging in these behaviors at a higher rate at the end of the sample than lower income individuals.

We need to be mindful of this pattern in our analysis. For instance, if we do not account for the initial levels at which respondents engaged in these behaviors, we categorize people as not increasing their social distancing behaviors when, given our definition, it was not possible for them to do so. Any findings under this approach could be biased, potentially reflecting differences by income in pre-pandemic behavior versus pandemic-induced adoption of self-protective behavior. Thus, to account for the level of activities before the pandemic started, we remove individuals for whom it was not possible to exhibit an above-median increase in social distancing behaviors.^16^ This results in a drop of 137 individuals from our analysis sample. Moreover, we find evidence that keeping these individuals in the sample does bias results towards finding stronger income gradients since dropped individuals tend to be in lower income quintiles (in part reflecting that they are older). Having dropped these individuals, we are left with an analysis sample consisting of respondents who were engaged in enough social activities that they could have an above-median shifts in behavior in response to the pandemic. We argue that the costliness of adopting these behaviors varies across income and other observed socioeconomic factors. In the next section, we demonstrate that people in lower income quintiles do indeed face substantial burdens that make behaviors such as social distancing more costly relative to members of higher income quintiles, which helps to explain why they adopt them less often.

## Results

### Connecting socio-demographic characteristics to income

The first panel of Table 2 summarizes the difference in means of several characteristics by income groups. Here, we have defined high income as the top three quintiles and low income as the bottom two quintiles. We find significant differences for most of these characteristics between high-income and low-income respondents. For example, non-white respondents were more likely to be low income than white respondents. Low-income respondents were slightly less likely to have a pre-existing health condition than those with high incomes. Of note, we find no significant income differences for beliefs in the effectiveness of social distancing. We also see that lower income people have a significantly smaller probability of increasing two of the self-protective behaviors we consider at the 5% level, while the other is significant at the 10% level. The second panel compares the difference in means between members of the third income quintile and the fifth quintile. Many of the differences that exist between income groups in the first panel persist here. However, unlike the first panel, we no longer see significant differences along individual characteristics such as race, access to open air at home, working part-time, continuing to work and lost income. When looking at behaviors we find no significant difference between middle- and high-income people increasing social distancing and increased hand washing or mask wearing behaviors. We still see a difference in the amount of people in each group changing any behavior. This is consistent with our discussion of the relationship between income and behavior in Fig. 1. We draw two conclusions from this table. First, there are significant demographic and behavioral differences between low- and high-income individuals in our sample. Second, while there are fewer differences between middle- and high-income respondents, significant differences along some demographics and one of our behaviors persist. These findings suggest that policymakers need to pay attention to lower income populations to ensure they engage in self-protective behaviors. Addressing income or job losses could be useful ways to encourage more widespread adoption of self-protective behaviors.

**Table 2:** Differences in means by characteristic and income group

Characteristic	1				2			
	Low	High	Diff.	*p* value	Middle	High	Diff.	*p* value
Socio-demographics								
Non-White	0.20	0.12	0.08	0.00	0.13	0.11	0.02	0.51
Male	0.31	0.52	− 0.20	0.00	0.40	0.68	− 0.28	0.00
New York-California	0.47	0.57	− 0.11	0.00	0.44	0.69	− 0.25	0.00
Elderly	0.47	0.35	0.12	0.00	0.43	0.22	0.21	0.00
Health								
Pre-existing condition	0.43	0.46	− 0.03	0.40	0.48	0.42	0.06	0.21
Housing								
Urban	0.37	0.43	− 0.06	0.05	0.29	0.62	− 0.34	0.00
Home w/o open air access	0.24	0.10	0.15	0.00	0.12	0.10	0.02	0.48
Elderly exposure	0.49	0.44	0.05	0.11	0.51	0.37	0.14	0.01
Work arrangements and losses								
Working	0.59	0.77	− 0.18	0.00	0.69	0.85	− 0.16	0.00
Full-time | working	0.23	0.66	− 0.43	0.00	0.54	0.82	− 0.29	0.00
Part-time | working	0.20	0.11	0.09	0.00	0.15	0.09	0.06	0.12
Can work from home | working	0.76	0.84	− 0.08	0.01	0.76	0.91	− 0.15	0.00
Stopped working | working	0.58	0.30	0.28	0.00	0.40	0.15	0.25	0.00
Tele-working | working	0.11	0.44	− 0.33	0.00	0.27	0.58	− 0.3	0.00
Still working | working	0.24	0.18	0.06	0.07	0.22	0.16	0.05	0.22
Mean lost HH. income ($1000)	0.40	0.51	− 0.11	0.00	0.46	0.55	− 0.09	0.07
Beliefs and perceptions								
Social distancing effectiveness	0.70	0.75	− 0.05	0.10	0.73	0.78	− 0.05	0.27
Local infection rate	0.36	0.44	− 0.08	0.01	0.32	0.51	− 0.18	0.00
Benefits from pandemic	0.76	0.89	− 0.13	0.00	0.85	0.90	− 0.05	0.12
Outcomes								
Changed behavior	0.81	0.92	− 0.11	0.00	0.89	0.95	− 0.06	0.02
Increased SD	0.50	0.57	− 0.07	0.06	0.56	0.58	− 0.02	0.75
Increased hand washing-mask wearing	0.63	0.76	− 0.13	0.00	0.77	0.77	0.00	0.98
*N*	1006				406			

This table contains the results from difference of means tests for the listed characteristics. The calculated means are for the proportion of people that are low income vs high income and medium income vs high income. For the first panel low income is defined as the bottom two income quintiles and high income is defined as the top three quintiles. For the second panel, middle income is defined as the third quintile and high income the fifth quintile. The “Diff.” column is the difference of these means and the *p* value associated with the difference

**Fig. 4:**
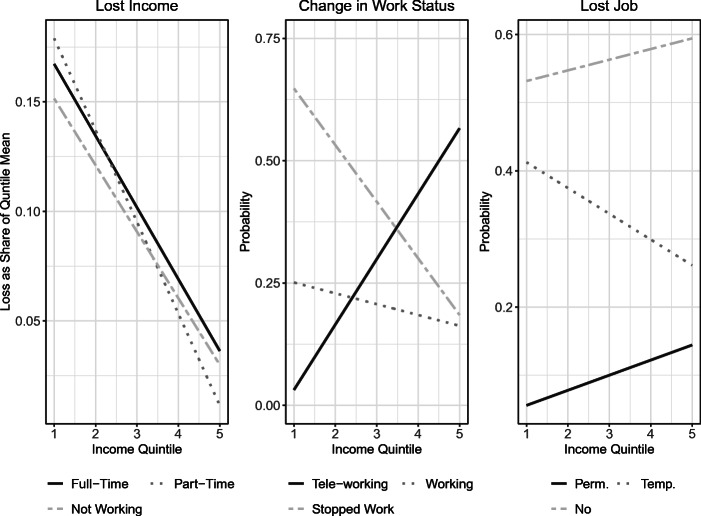
Realized losses and labor market changes by income: Realized income losses by income quintile. Income losses are normalized by the mean income of the respondent’s quintile. Changes in work status are the proportion of respondents within an income quintile that were observed working within the sample. Job losses are proportions of each type of labor transition within an income quintile. To improve readability, we have omitted the markers for the quintile means and display the linear trend. These trends were tested for statistical significance

**Fig. 5:**
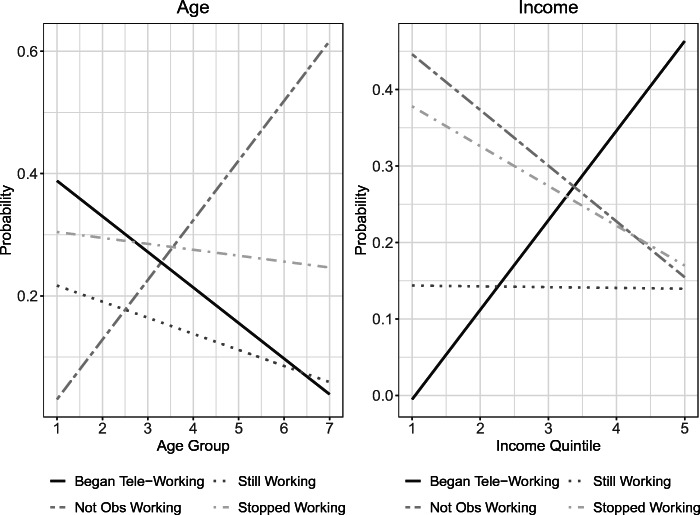
Probability of work arrangement by age group and income quintile: Proportion of respondents within an age group or income quintile that are classified into our work arrangements variable. To improve readability, we have omitted the markers for the quintile means and display the linear trend. These trends were tested for statistical significance

**Fig. 6:**
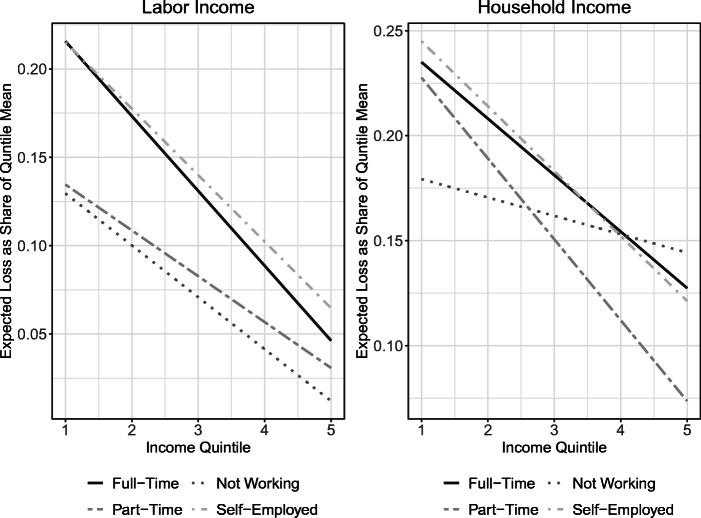
Expected income losses by income and labor status: The left panel summarizes expected labor income losses across income quintiles and labor statuses (i.e., working full-time, part-time, self-employed, or not working at all). The right panel summarizes expected household income losses along the same two dimensions. In both panels, expected losses are normalized by the mean income of the respondent’s quintile. To improve readability, we have omitted the markers for the quintile means and display the linear trend. These trends were tested for statistical significance

Figure 6 in the Appendix explores expected losses to labor and household income by labor status and income quintile. Expected losses to labor income are a much larger share of income for low-income respondents. For example, people in the first income quintile reported expected labor income losses of over 10% while respondents in the fifth income quintile expected losses of no more than 5%. We observe a similar pattern when looking at expected household income losses. First quintile expected losses range from nearly 20% to about 25% while fifth quintile losses range from 10% to just under 15%. We also find that the difference between the mean expected labor income loss for the first and fifth income quintile is statistically significant. Figure 4 assesses losses that have already occurred. We observe a similar relationship between income quintile and the magnitude of income losses in the first panel. The second panel examines changes in work status. We see that transitions to tele-working rise with income. We observe the reverse pattern for low-income people, who were most likely to have stopped working altogether. The third panel further examines job losses due to the pandemic. We find that the lowest income respondents had the least amount of job security and the highest probability of temporary unemployment. An interesting pattern is that higher income individuals were the most likely to permanently lose their jobs, despite also having the highest level of job security across all income quintiles. This may reflect selection: higher income jobs are more secure in general so a job loss reflects a large and permanent shift, e.g., a bankruptcy. Similar to expected income losses, the difference between mean household income losses for the first and fifth income quintile was statistically significant. Taken together, these figures demonstrate how the burdens of the pandemic have fallen especially hard on the lower income quintiles. These are the people who have lost relatively more income and employment, which may make it harder for them to engage in self-protective behaviors.

**Fig. 7:**
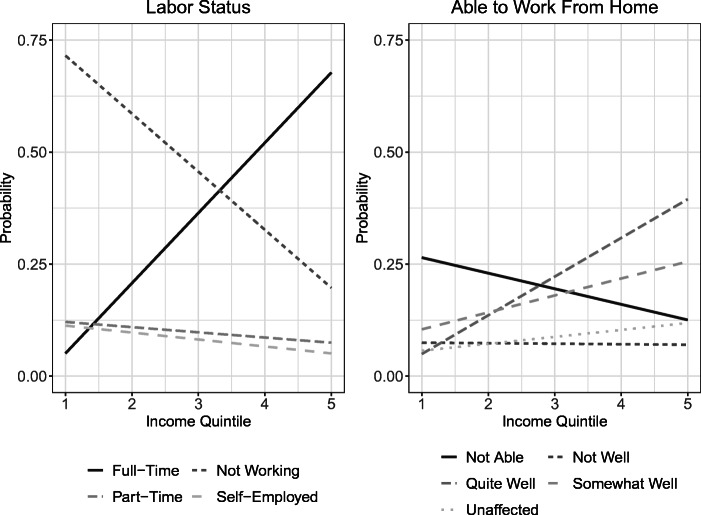
Probability of labor characteristic by income quintile: This figure shows the proportion of respondents within each income quintile that have different labor market characteristics. The left panel shows the work status of respondents (i.e., working full-time, part-time, self-employed, or not working at all). The right panel depicts how well a respondent can work from home. The share of respondents that are not working (and thus unable to work from home altogether) are omitted. To improve readability, we have omitted the markers for the quintile means and display the linear trend. These trends were tested for statistical significance

Next, we consider work arrangements by income. Figure 7 in the Appendix consists of two panels, which plot labor status (full-time, part-time, self-employed or not working) and how well they can work from home, respectively. According to the figure, full-time employment and the ability to work from home rise with income. Lower income people are more likely to report either that they stopped working or that they experienced no change in work status (which includes not having switched to tele-working). For example, nearly 75% of respondents in the fifth income quintile are working full-time. About the same percentage of respondents in the first quintile are not working. From the center panel of Fig. 4, we see that more than 50% of respondents in the fifth income quintile reported transitioning to tele-work post-pandemic, whereas only 10% of those in the first quintile did so. This pattern appears consistent with the fact that nearly 40% of low-income respondents report being unable to work from home.^17^

The other broad categories of socio-demographic characteristics that are potentially associated with income and behavior changes are pre-existing health conditions, household features and beliefs related to the pandemic. From Table 1 we know that 45% of survey respondents reported having a pre-existing health condition such as diabetes, high blood pressure, heart disease, or asthma. Table 3 breaks down the prevalence of specific pre-existing conditions by income quintile. Given strong income-health gradients found in other literature, it is surprising that we do not find a meaningful relationship between income and most health conditions. For instance, we find higher proportions of diabetes among higher income people in our sample. This may reflect that the sample is not representative of the USA in terms of the relationship between income and health.

**Table 3:** Pre-existing health conditions by income

Income	Diabetes	High blood pressure	Heart disease	Asthma
First quintile	0.14	0.30	0.06	0.13
Second quintile	0.14	0.32	0.05	0.12
Third quintile	0.19	0.32	0.05	0.14
Fourth quintile	0.16	0.28	0.05	0.15
Fifth quintile	0.25	0.21	0.05	0.14

This table summarizes the share of respondents with various pre-existing health conditions that fall into each income quintile for the US sample. Respondents may have multiple conditions

In contrast, housing is strongly related to income. As seen in Table 4, higher income respondents are far more likely to live in urban areas (versus suburban or rural), homes (versus apartments), and have access to open air where they reside compared to lower income respondents. These housing characteristics influence the cost of self-protective behaviors. For example, larger home types or having access to open air can make it easier to maintain social distancing. This is an area where policy actions now could alleviate the harm from future pandemics. Increasing access to open air in public housing or parks could be effective ways to address the behavior discrepancies we observe between low- and high-income respondents. As we noted earlier, we do not observe a pattern between income and beliefs in the effectiveness of social distancing.^18^ Across all income quintiles, 70–78% of respondents believe social distancing is either very effective or extremely effective. This suggests policymakers have been successful in sharing some information about the pandemic. However, we will later discuss that there is a gap between these beliefs and the adoption of self-protective behaviors across income groups.

**Table 4:** Home characteristics by income

Variable	First	Second	Third	Fourth	Fifth
Current home area					
Urban	0.41	0.33	0.29	0.41	0.62
Semi-urban/residential	0.41	0.52	0.60	0.49	0.35
Countryside	0.18	0.15	0.11	0.10	0.03
Total	168	190	210	243	196
Current home type					
House	0.43	0.56	0.63	0.78	0.77
Apartment	0.39	0.29	0.24	0.15	0.18
Condominium	0.03	0.07	0.07	0.04	0.05
Trailer	0.10	0.07	0.04	0.01	
Shelter	0.02				
Other	0.03	0.02	0.01	0.02	0.01
Total	168	190	210	243	196
No open air access					
Not selected	0.67	0.83	0.88	0.92	0.9
Selected	0.33	0.17	0.12	0.08	0.1
Total	168	190	210	243	196

This table summarizes the share of respondents within each income quintile broken out by different categories of home characteristics for the US sample. Shares are calculated within each of the three categories. The total values are the total number of respondents within each income quintile

**Fig. 8:**
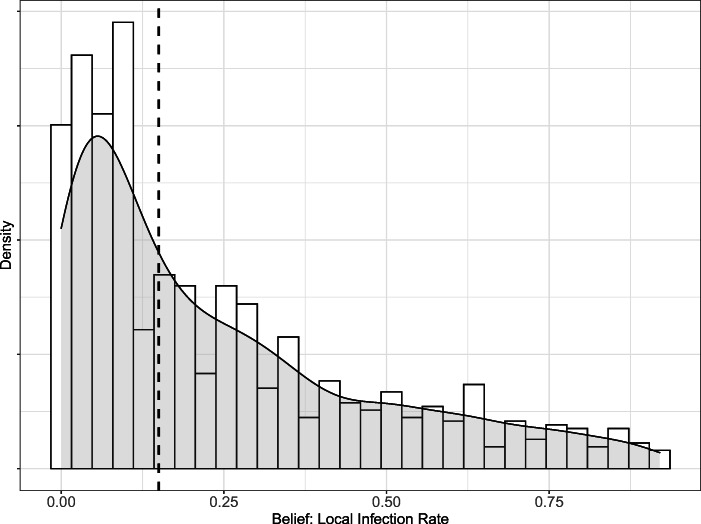
Distribution of beliefs about local infection rates: Distribution of the reported beliefs of respondents about the rate of infections within their local community. The raw data are presented in the bars, while the density of these beliefs is overlaid. The dashed line represents the median value for the local infection rate

The survey data also contain respondents’ beliefs about various rates related to the disease such as infection rates, likelihood of contracting the disease, and so on. However, it is difficult to interpret responses. For example, consider beliefs about the local infection rate. The distribution of these beliefs is presented in Fig. 8. We can see that many respondents report implausibly small and large numbers. This could reflect several factors, including misinformation about the spread of illness, difficulties with probabilistic thinking, which is well documented in the literature (see, e.g., Barth et al. 2020; Lillard and Willis 2001; Delavande et al. 2006), fatalistic beliefs (e.g., Akesson et al. 2020), optimism about herd immunity, etc.^19^ Another possibility is that misinformation about the pandemic initially spread by the federal government and news media may have “spoiled” the way people link less direct beliefs to outcomes. This in part motivates why we opt to include beliefs about the effectiveness of social distancing to ensure that people who believe these behaviors are effective are also the people engaging in them more frequently. In any case, these interpretational difficulties will limit conclusions we can draw using some of the beliefs variables.

### Factors associated with behavior change

Our analysis until now shows that several factors are related to income. These factors could potentially help to explain differences across income groups in self-protective behaviors depicted in Fig. 1. In this section, we explore which socio-demographic characteristics are associated with behavior changes. We begin by providing summary statistics and then discuss and analyze findings from our main estimates from regressions of behavior change onto different sets of explanatory variables.

Table 5 summarizes the difference in means across various characteristics for those who increased social distancing behavior according to our metric. We find significant differences between males and females and between those who believe in the effectiveness of social distancing. These findings suggest that women are more likely than men to increase social distancing as are those who believe strongly in the effectiveness of social distancing. We see a significant difference between those who increased social distancing behaviors and work statuses. Specifically, we see that respondents who continued to work (as opposed to stopping work or transitioning to tele-work) engaged in increased social distancing at a lower rate than those who had transitioned to another work arrangement. We also see that respondents in New York and California were more likely to increase social distancing behaviors than individuals from Texas or Florida, which may be explainable by political factors. Older respondents also increased social distancing more than younger respondents. This pattern appears consistent with the elevated risk elderly people face from Covid-19. Another significant difference can be seen between respondents without access to open air at home, which we view as a barrier towards adopting this self-protective behavior. We do not find significant differences across other individual characteristics. Finally, we also see that people increasing social distancing behaviors have a significantly larger probability of increasing other self-protective behaviors. This finding is reassuring, as we would expect to see co-movement among the various measures we consider.

**Table 5:** Difference in means by characteristic for increased social distancing

Characteristic	Yes	No	Diff.	*p* value
Socio-demographics				
Non-White	0.13	0.16	− 0.03	0.26
Male	0.36	0.53	− 0.18	0.00
New York-California	0.58	0.49	0.10	0.00
Elderly	0.43	0.34	0.10	0.00
Health				
Pre-existing condition	0.43	0.44	− 0.01	0.87
Housing				
Urban	0.37	0.43	− 0.06	0.11
Home w/o open air access	0.08	0.17	− 0.08	0.00
Elderly exposure	0.49	0.43	0.06	0.08
Work arrangements and losses				
Working	0.68	0.76	− 0.08	0.01
Full-time | working	0.52	0.57	− 0.05	0.26
Part-time | working	0.15	0.13	0.03	0.33
Can work from home | working	0.81	0.82	− 0.01	0.74
Stopped working | working	0.39	0.36	0.03	0.49
Tele-working | working	0.38	0.33	0.05	0.24
Still working | working	0.14	0.24	− 0.1	0.00
Mean income quintile	0.48	0.43	0.05	0.16
Mean lost HH. income ($1000)	0.49	0.51	− 0.02	0.63
Beliefs and perceptions				
Social distancing effectiveness	0.84	0.64	0.20	0.00
Local infection rate	0.35	0.45	− 0.11	0.00
Benefits from pandemic	0.87	0.85	0.03	0.27
Outcomes				
Changed behavior	0.95	0.85	0.10	0.00
Increased hand washing-mask wearing	0.92	0.53	0.39	0.00
*N*	824			

This table contains the results from difference of means tests for the listed characteristics. The calculated means are for the proportion of people that increased social distancing behaviors versus those that did not increase these behaviors. The Yes column is the average value of the characteristic for those that increased social distancing. The No column is the average value of the characteristic among those who did not increase social distancing. The “Diff.” column is the difference of these means and the *p* value associated with the difference

For our main analysis, we examine three outcomes: any behavior change, social distancing, and mask wearing or hand washing. As mentioned earlier, while we examine three different outcomes, we are testing one main hypothesis: whether socio-demographic factors predict the adoption of self-protective behaviors. We view using three measures of behavior as important. First, each of these behaviors impose a different cost for individuals. Different types of people may be more responsive to low-cost behaviors such as changing behaviors or hand washing-mask wearing as opposed to more costly behaviors such as social distancing. Second, as these behaviors are correlated with one another, common findings across each of these behaviors serves as a form of robustness for the demographic patterns we identify.

For each outcome, we estimate linear probability models as a function of income and different sets of explanatory variables. We use heteroskedastic robust standard errors. Our main findings are summarized in Table 6. We discuss these results and other findings in greater detail in the following subsections. In general, we find that income, work arrangements such as tele-working, lost income and beliefs about the effectiveness of social distancing are significantly associated with the self-protective measures we examine. Detailed results are presented in Tables 7, 8 and 9 in the Appendix. In each table, all columns include income quintiles as explanatory variables. Column (1) includes only income, column (2) adds in socio-demographic characteristics, column (3) adds in pre-existing health conditions, column (4) brings in housing characteristics, column (5) introduces work arrangements and economic loss characteristics, and column (6) adds in beliefs about social distancing and local infection rates and perceived benefits from the pandemic. Finally, in column (7) we include all of these sets of controls in a single specification. We will discuss each of these columns in the following subsections.^20^

**Table 6:** Summary of factors associated with self-protective behaviors

Variable	Metric	CB	SD	WB	Variable	Metric	CB	SD	WB
Income	Significant	✓	✓	✓	Countryside	Significant	✗	✗	✗
	Sign	**+**	**+**	**+**		Sign	**NA**	**NA**	**NA**
Non-White	Significant	✗	✗	✓	No access to open air	Significant	✗	✓	✗
	Sign	**NA**	**NA**	**+**		Sign	**NA**	**–**	**NA**
Male	Significant	✗	✓	✗	Not working	Significant	✗	✗	✓
	Sign	**NA**	**–**	**NA**		Sign	**NA**	**NA**	**+**
Florida	Significant	✗	✓	✗	Tele-working	Significant	✓	✓	✓
	Sign	**NA**	**–**	**NA**		Sign	**+**	**+**	**+**
New York	Significant	✗	✗	✗	Not working pre-Covid	Significant	✗	✓	✗
	Sign	**NA**	**NA**	**NA**		Sign	**NA**	**+**	**NA**
Texas	Significant	✗	✓	✗	Lost income	Significant	✓	✓	✓
	Sign	**NA**	**–**	**NA**		Sign	**+**	**+**	**+**
56 or older	Significant	✗	✗	✓	Effectiveness of SD	Significant	✓	✓	✓
	Sign	**NA**	**NA**	**+**		Sign	**+**	**+**	**+**
Pre-existing condition	Significant	✓	✓	✓	Area infection rate	Significant	✗	✓	✓
	Sign	**+/–**	**–**	**+**		Sign	**NA**	**–**	**–**
Semi-urban/residential	Significant	✗	✗	✓	Benefits	Significant	✓	✓	✓
	Sign	**NA**	**NA**	**–**		Sign	**+**	**+**	**+**

This table summarizes the findings from linear probability models examining the association between socio-demographic characteristics and three different self-protective behaviors. Detailed tables with coefficient estimates, standard errors, significance levels and other controls can be found in the Appendix. “CB” stands for changed behaviors, “SD” stands for increased social distancing behaviors, and “WM” stands for increased hand washing or mask wearing

#### Income

Across all three of our dependent variables we find strong, statistically significant associations with income. Higher income individuals are more likely to engage in the behaviors we examine. To fix ideas, relative to the first income quintile, a member of the fifth income quintile is 10–15 percentage points more likely to change their behaviors, 11–24 percentage points more likely to increase social distancing behaviors, and 17–25 percentage points more likely to increase hand washing or mask wearing. Put another way, when all controls are included, a member of the fifth income quintile is 13% more likely to change their behaviors, 32% more likely to increase social distancing and 30% more likely to increase hand washing or mask wearing. We find that these income effects are fairly robust to the inclusion of controls. From the baseline to the case where we include all of our controls, the size of the coefficient estimates remain fairly stable as we add additional variables, which means that these other factors do not fully explain the income gradient. The slight exception is for the increased social distancing outcome. When all of our controls are added, we only see a significant difference between the fifth and the first income quintiles, suggesting that our explanatory variables help to explain the relationship between income and what appears to be the a costly self-protective measure. In general, the income gradients presented here strongly suggest that the adoption of self-protective behaviors is a costly prospect, one that is easier for people with more income. While providing cash transfer could help, the income gradients alone do not provide very much policy guidance. Thus, we now consider whether additional factors associated with self-protective behavior adoption.

#### Gender, age, race and location

The next set of control variables we examine are gender, age, race and state. We do not find many significant associations between these factors and the change behaviors outcome. In the baseline case we see negative associations between males, people 56 years or older, and some regional effects. Some of these relationships lose significance when other variables are added to the analysis, though we find more robust patterns when examining increases in social distancing behavior. We find strong negative associations between males, and respondents from Florida and Texas, which maintain significance once other controls are added. To fix ideas, we find that males are 23% less likely than females to increase social distancing. This could be evidence that the pandemic is driving women into “traditional” care taker roles—staying at home to maintain the household—while males continue to work in person and are unable to adopt social distancing behaviors. Similarly, we find that relative to respondents from California, people in Texas and Florida tended to be 21% and 22% less likely to increase social distancing, respectively. These results may presage the surges in Covid-19 cases that happened in these two states that began towards the end of June 2020. This finding complements recent work that has found a significant relationship between political affiliation and beliefs about the Covid-19 pandemic and the adoption of social distancing behaviors. These are states led by governors who were less responsive to the outbreak of the pandemic, which may have had an influence on behavior. Finally, we find positive significant associations between race and people 56 years or older for the hand washing-mask wearing outcome. Specifically, we find that Black respondents are 19% more likely than white respondents to increase hand washing or mask wearing.^21^ We find a similarly sized relationship for those 56 years or older. It is interesting that find these effects for increased hand washing or mask wearing. This may reflect the fact that of the three activities we examine, this one is a relatively low-cost way to self-protect for people who face risks, but are unable to engage in higher cost, less practical activities, such as social distancing.

#### Health

We also examine various pre-existing health conditions, including diabetes, high blood pressure, heart disease, asthma, allergies, and other conditions. Overall, and surprisingly, these variables are not strongly correlated to behavior change. Oddly, we find a strong *negative* association between heart disease and increased social distancing, which may reflect that people with heart disease are generally unhealthy and thus less likely to engage in self-protective behaviors. Yet, it is surprising that health conditions more strongly associated with serious illness (e.g., diabetes, asthma, or high blood pressure) are not associated with behavior change. An exception is that we find a robust significant association between allergies and increases in hand washing and mask wearing. Allergies would presumably be less likely to be associated with unobserved factors capturing an unwillingness or inability to engage in self-protective behaviors. Another possibility that people with allergies could feel they are becoming ill even if they are not and thus be more willing to take precautions. More generally, the lack of a health gradient could be a byproduct of our non-representative sample. As mentioned previously, pre-existing conditions were not targeted for representativeness either in the data collection process. As our understanding of Covid-19 grows, future data collection efforts may want to target specific health conditions to determine if an association with self-protective behaviors exists.

#### Housing

Next we examine housing characteristics. We find a negative significant relationship between respondents in the countryside and changing behaviors but this association becomes indistinguishable from zero as other controls are added. We find a robust negative association for having no access to open air space at home and increased social distancing behavior. In our full control case, we find that respondents that live in homes without open air access are 20% less likely to increase social distancing behaviors. We find this to be an intuitive result. People who are more comfortable sheltering-in-place are more likely to do it. Policies aiming to slow the pandemic should take these factors into account as they suggest cramped and uncomfortable housing can potentially undermine efforts to “flatten the curve.” This result could also guide the design of future housing policy as government prepare for future pandemics. For example, communities could increase the size and availability of public parks to accommodate social distancing. Governments could also prioritize the opening of parks or other open public spaces during a pandemic, though of course the risks in terms of increased exposure would need to be weighed against the benefits in terms of higher rates of social distancing among low-income people. Another, longer run possibility would be to incorporate some open air spaces such as balconies or community gardens into the designs for public housing, which would help facilitate social distancing behavior. Finally, we find similar patterns for the two other outcome variables, though estimates are less precise in the final specification.

#### Work arrangements and losses

We also consider work arrangements and economic losses. In general we find fairly consistent results across all three of our outcome variables. People who transitioned into tele-working are more likely to change behaviors, increase social distancing, and increase hand washing-mask wearing. This association ranges from roughly 9–15 percentage points relative to somebody who continued to work. When all controls are included, a person that transitions to tele-work is 20 to 28% more likely to increase these self-protective behaviors. This effect is robust to the inclusion of other controls. We find a similarly sized effect for those that stopped working or never worked but significance was retained with less consistency. This result is intuitive. People who can work from home are more likely to abide by stay at home orders. Factors related to work arrangements, which vary across socio-demographic groups, can determine the sustainability and effectiveness of policies aiming to prevent the spread of illness.^22^ From a policy perspective, governments could offer incentives or resources for firms to increase the availability of tele-work to their employees. Beyond preparing for future pandemics, this policy may also help firms compete for talent along non-monetary dimensions. For example, it appears that tele-medicine may remain after the Covid pandemic (Smith et al. 2020). Of course, this type of policy has its limits as some work, by its nature, requires physical contact.

We also find that realized household income losses have a significant positive association with each of these behaviors. After controlling for extreme lost income values, we find that for every $1,000 lost a respondent is 1–4 percentage points more likely to adjust each of the behaviors we examined. People who have experienced these losses have already been harmed by the pandemic. As a result, they may be more careful than others and view contracting the disease as a higher risk. Another possibility is that these people have fewer monetary resources and may not have money to cover medical expenses if they were to contract the disease. This speaks to the importance of polices that provide direct monetary relief to people during a pandemic. Our results suggest that policies such as the CARES Act, which featured direct compensation to all citizens is an effective way to promote the adoption of self-protective behaviors. Other studies of the CARES Act have shown it was a useful short-term policy to help people smooth their consumption during the pandemic (Carroll et al. 2020).

#### Beliefs and perceptions

The final set of variables we examined were beliefs and perceptions about the pandemic. Reassuringly, we find a fairly consistent effect for beliefs in the effectiveness of social distancing across the three behaviors we included. These findings are strongest for the changed behaviors and increase social distancing variables. We find similar results but with weaker significance for increased hand washing-mask wearing. Yet, there is a disconnect between these beliefs and individual behavior. For example, approximately 97% of respondents in the first and fifth income quintiles believe social distancing is effective. However, 80% of people in the first income quintile reported changing any behavior while 93% people in the fifth income quintile reported any behavior change. We see a similar discrepancy when looking at increases in social distancing behaviors: 45% of respondents in the first quintile and 57% in the fifth income quintile. This leads us to the somewhat depressing conclusion that is, however, entirely in line with our results: many people from lower income groups recognize the effectiveness of self-protective measures, but do not adopt them, suggesting different costs of doing so compared to higher income people. For example, low-income people tend to not have access to tele-working, which affords people the opportunity to maintain social distancing without having to sacrifice labor income.

**Fig. 9:**
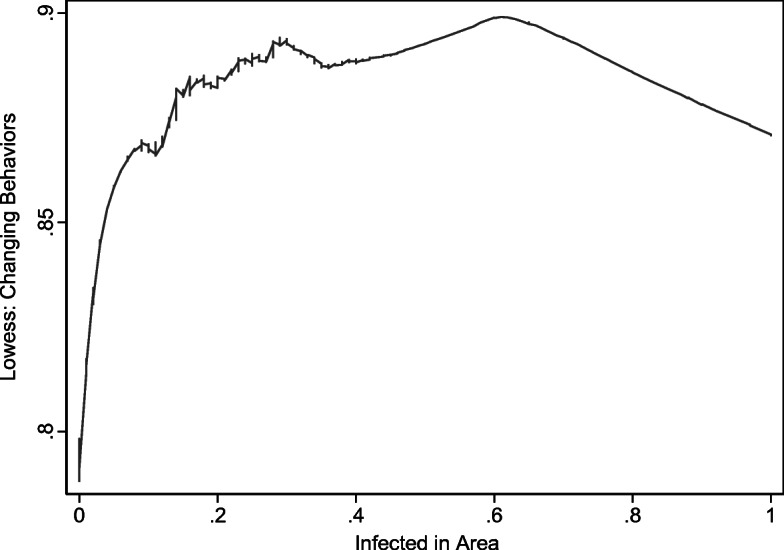
Lowess smoother changed behaviors: Plots the application of the Lowess smoother to the relationship between the binary changed behaviors outcome and beliefs about the local infection rate for the US analysis sample

**Fig. 10:**
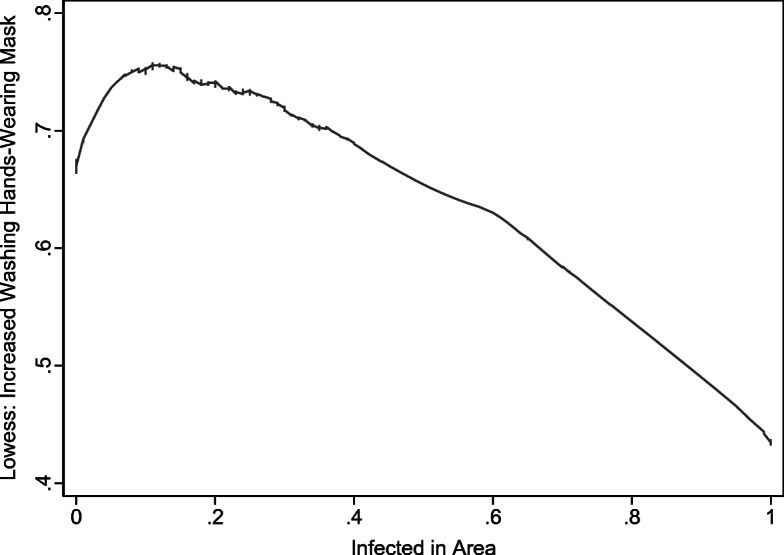
Lowess smoother increased social distancing: Plots the application of the Lowess smoother to the relationship between the binary increased social distancing outcome variable and beliefs about the local infection rate for the US analysis sample. Increased social distancing is defined as an above-median increase in self-protective behaviors (e.g., visiting public spaces, visiting friends and family) from before the pandemic to a few weeks after it started

**Fig. 11:**
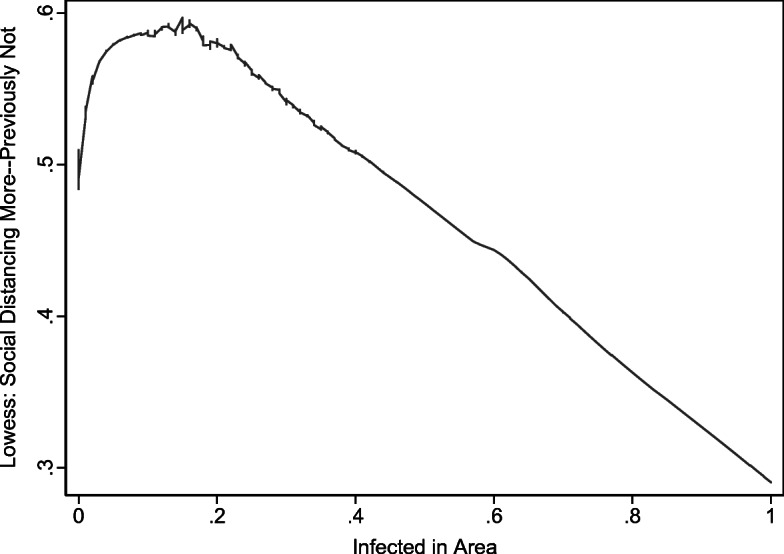
Lowess smoother increased hand washing-mask wearing: Plots the application of the Lowess smoother to the relationship between the binary increased hand washing-mask wearing outcome variable and beliefs about the local infection rate for the US analysis sample. Increased hand washing-mask wearing is defined as an above-median increase in hand washing or mask wearing behavior from before the pandemic to a few weeks after it started

One finding that surprised us was the negative association between beliefs about local infection rates and increases in self-protecting behaviors. As discussed previously, the distribution of respondent beliefs about local infection rates has significant mass at the low and high end, which are difficult to reconcile with reality. In Figs. 9, 10, and 11 we present Lowess smoother results for three behavioral outcomes of interest and this belief.^23^ In each case, people who reported an infection rate of 20% or fewer exhibit the expected response: a rise in perceived infection rates is associated with more protective behavior. Thus, negative coefficient estimates are driven by people with implausibly high perceptions of infection rates. This could reflect respondent confusion. It could also reflect a sort of fatalism, i.e., people believe infection rates are so high that they are bound to become infected, too, and thus don’t bother to engage in protective behaviors. Fatalism is a well-documented phenomenon in several fields (see, e.g., Akesson et al. 2020; Ferrer and Klein 2015; Shapiro and Wu 2011). These findings suggest that pockets of misinformation persist within the population. It is crucial that policymakers provide accurate and complete information about the risks of the pandemic and how people can best protect themselves from infection.

We also find some positive associations between perceived benefits from the pandemic and increases in our behaviors of interest. Less pollution and more family time were two that came out as significant and tended to retain significance as other controls were added. In Appendix Table 11, we present cross-tabulations of the survey data which indicate most of the people identifying these benefits belonged to higher income quintiles.

### Robustness checks

We conduct a series of robustness checks to these specifications. The results for these analyses are available in the Supplemental Appendix of Papageorge et al. (2020). First we consider whether a respondent was engaging in these self-protective behaviors at all following the start of the pandemic. We also look at whether there were distinct behavior differences between those that had experienced a loss due to the pandemic and the pooled sample. In another test examine each state individually and pooled groups of states. In general, these analyses align with our main results. We also consider the intensive margin for increased social distancing and hand washing or mask wearing behaviors. A respondent’s income and beliefs about the effectiveness of social distancing did not have a significant association with larger increases in either of these self-protecting behaviors. Other effects are consistent with our main analysis. Finally, as mentioned previously, the data collected by Belot et al. (2020b) do not contain information on educational attainment. We use information on a respondent’s profession to construct a proxy for whether they have a college degree and incorporate it into our specifications.^24^ We find that education is positively associated with increases in self-protective behaviors. The inclusion of this variable does not appreciably alter our other findings.

## Conclusion

The Covid-19 pandemic has shaken the world. The USA in particular has struggled with its response immensely and is engaged in a contentious debate about the best path forward. The debate often focuses on the strictness of safety measures to balance public health concerns with economic costs. Often absent from the debate is that any policy will have unequal consequences for different socioeconomic groups and, moreover, that different groups respond differently to incentives. In this paper we examined how socio-demographic factors predict the adoption of protective behaviors during the Covid-19 pandemic in the USA using unique survey data collected during the third week of April 2020 by Belot et al. (2020b). This collection effort was specifically tailored to the pandemic, enabling us to incorporate unique variables such as work status changes and beliefs into our analysis.

We examined three different self-protective behaviors (i) whether the respondent changed any behavior, (ii) whether the respondent increased social distancing behaviors and (iii) whether the respondent increased hand washing or mask wearing behaviors. While these behaviors varied in terms of the costs imposed on individuals, they are all correlated and informative about which types of people are responding to the pandemic. We find that income, work arrangements such as tele-working, lost income and beliefs about the effectiveness of social distancing are significantly associated with the self-protective measures we examine. These findings are generally robust to the inclusion of several controls and sample adjustments. Our results highlight the heaviest burden of these measures are placed on those who are already suffering the most from the pandemic. For instance, members of the first income quintile, who have endured substantial monetary losses, are less likely to have access to tele-working, forcing them to choose between their health and a paycheck. More broadly, we have demonstrated that many of the socioeconomic health gradients present in other settings are also present during the Covid-19 outbreak. This information provides crucial insights into the real world implications of the current pandemic and ought to inform policymakers as they respond to the latest resurgence of the disease.

Our analyses highlight many areas for policymakers to address behavioral differences and prepare for future pandemics. First, increasing access to open air could reduce the cost of adopting behaviors such as social distancing. One way to achieve this end could be the prioritization of opening more public parks either before a pandemic or when designing lockdown measures. Another is to revisit the design of public housing to include open air spaces such as balconies or community gardens. Second, increasing access to tele-work for more employees will lower the burden of self-protective behaviors. Governments could either incentivize firms to offer this work arrangement or provide them with resources to make it available. Firms may also benefit from this measure as it improves their ability to attract talent in labor markets. Third, governments can offer direct monetary relief to citizens to ease the burdens of the pandemic. This policy can insulate people from seeking additional work that may prevent them from following self-protective behaviors. Finally, existing long-term data collection efforts should incorporate questions with an eye towards future public health crises. This could take many forms but some examples include information about work arrangements and beliefs in government experts and institutions.

While many of the questions raised and discussed in this paper focus on a specific point in time, the Covid-19 pandemic will eventually run its course. However, it would be shortsighted and naive to think that another virus, perhaps an even more damaging one, will not come about in the future. Indeed, some specialists believe that this virus will be cyclical, returning annually. If so, the questions we are addressing now will be important not only as we move through the current crisis, but also as we begin to prepare for the next one. Social scientists who study behavior—and the policies that affect it—must play a critical role in these efforts. One way is through the collection and analysis of new survey data, which shed light on what behavior can be expected of different segments of the population during a pandemic given heterogeneity in the incentives, constraints and circumstances people face. These data could be used not only to describe behavior, but also in more targeted research projects, such as: examining how information is transmitted and beliefs about the pandemic are formed and affect behavior, analyzing location-specific policy responses and their relative merit, and calibrating epidemiologically grounded models relating variation in individual behavior to the spread of illness, among many others.

## Appendix. Additional figures and tables

### A.2 Tables

**Table 7:** Factors associated with changing behaviors

	(1)	(2)	(3)	(4)	(5)	(6)	(7)
Income							
Second quintile	− 0.00	− 0.00	− 0.01	− 0.01	− 0.01	0.00	− 0.00
	(0.04)	(0.04)	(0.04)	(0.04)	(0.04)	(0.04)	(0.04)
Third quintile	0.09^∗∗^	0.09^∗∗^	0.09^∗∗^	0.08^∗∗^	0.07^∗^	0.08^∗∗^	0.07^∗^
	(0.04)	(0.04)	(0.04)	(0.04)	(0.04)	(0.04)	(0.04)
Fourth quintile	0.11^∗∗∗^	0.12^∗∗∗^	0.11^∗∗∗^	0.10^∗∗∗^	0.09^∗∗∗^	0.10^∗∗∗^	0.09^∗∗^
	(0.04)	(0.04)	(0.04)	(0.04)	(0.04)	(0.04)	(0.04)
Fifth quintile	0.14^∗∗∗^	0.15^∗∗∗^	0.15^∗∗∗^	0.12^∗∗∗^	0.11^∗∗∗^	0.12^∗∗∗^	0.10^∗∗∗^
	(0.04)	(0.04)	(0.04)	(0.04)	(0.04)	(0.03)	(0.04)
Socio-demographics							
Black		− 0.04					− 0.02
		(0.04)					(0.04)
Other		− 0.04					− 0.05
		(0.05)					(0.05)
Male		− 0.06^∗∗^					− 0.03
		(0.02)					(0.02)
Florida		0.01					− 0.00
		(0.03)					(0.03)
New York		− 0.03					− 0.04
		(0.03)					(0.03)
Texas		− 0.05					− 0.04
		(0.03)					(0.03)
56 or older		− 0.05^∗∗^					− 0.02
		(0.02)					(0.03)
Health							
Diabetes			− 0.05				− 0.05^∗^
			(0.03)				(0.03)
High blood pressure			− 0.03				− 0.01
			(0.03)				(0.03)
Heart disease			0.03				0.02
			(0.05)				(0.05)
Asthma			0.04				0.04
			(0.03)				(0.03)
Allergies			0.04				0.02
			(0.02)				(0.02)
Other condition			0.06^∗∗^				0.06^∗∗^
			(0.03)				(0.03)
Housing							
Semi-urban/residential				− 0.03			− 0.03
				(0.02)			(0.02)
Countryside				− 0.08^∗∗^			− 0.05
				(0.04)			(0.04)
No access to open air				− 0.04			0.00
				(0.03)			(0.03)
Work arrangements-losses							
Stopped working					0.10^∗∗^		0.07
					(0.04)		(0.04)
Began tele-working					0.12^∗∗∗^		0.09^∗∗^
					(0.04)		(0.04)
Not observed working					0.07^∗^		0.04
					(0.04)		(0.04)
Other					0.18^∗∗∗^		0.13^∗∗∗^
					(0.04)		(0.04)
Flag extreme lost income					− 0.05^∗∗^		− 0.03
					(0.03)		(0.03)
Lost HH Inc./$1000 Orig					− 0.00		0.00
					(0.00)		(0.00)
Lost HH Inc./$1000 Adj.					0.02^∗∗∗^		0.01^∗∗^
					(0.01)		(0.01)
Beliefs-perceptions							
Slightly effective						0.24^∗∗^	0.23^∗∗^
						(0.10)	(0.10)
Moderately effective						0.25^∗∗∗^	0.24^∗∗^
						(0.10)	(0.10)
Very effective						0.33^∗∗∗^	0.32^∗∗∗^
						(0.09)	(0.09)
Extremely effective						0.37^∗∗∗^	0.34^∗∗∗^
						(0.09)	(0.09)
Infected in area						0.04	0.01
						(0.04)	(0.04)
More free time						0.02	0.01
						(0.02)	(0.02)
More family time						0.05^∗∗^	0.04^∗^
						(0.02)	(0.02)
Less pollution						0.06^∗∗∗^	0.06^∗∗^
						(0.02)	(0.02)
Less noise						− 0.03	− 0.03
						(0.02)	(0.02)
Other						0.02	0.00
						(0.05)	(0.05)
Observations	955	955	955	955	955	955	955
*R* ^2^	0.033	0.049	0.046	0.040	0.064	0.099	0.138

This table presents estimates from regressions of whether a respondent changed any behavior after the pandemic started and various individual characteristics. Column (1) only considers income. Column (2) adds socio-demographics to income. Column (3) adds pre-existing health conditions to income. Column (4) adds housing characteristics to income. Column (5) adds work arrangement and losses to income. Column (6) adds beliefs and perceptions about the pandemic to income. Column (7) includes all of these controls in a single specification

Heteroskedastic robust standard errors in parentheses

^∗^*p* < 0.1, ^∗∗^*p* < 0.05, ^∗∗∗^*p* < 0.01

**Table 8:** Factors associated with social distancing more—previously not

	(1)	(2)	(3)	(4)	(5)	(6)	(7)
Income							
Second quintile	0.07	0.09	0.06	0.03	0.08	0.04	0.05
	(0.06)	(0.06)	(0.06)	(0.06)	(0.06)	(0.06)	(0.06)
Third quintile	0.12^∗^	0.15^∗∗^	0.11^∗^	0.07	0.13^∗∗^	0.07	0.08
	(0.06)	(0.06)	(0.06)	(0.06)	(0.06)	(0.06)	(0.06)
Fourth quintile	0.10^∗^	0.14^∗∗^	0.10	0.06	0.14^∗∗^	0.09^∗^	0.10
	(0.06)	(0.06)	(0.06)	(0.06)	(0.06)	(0.05)	(0.06)
Fifth quintile	0.16^∗∗^	0.24^∗∗∗^	0.16^∗∗^	0.11^∗^	0.20^∗∗∗^	0.13^∗∗^	0.15^∗∗^
	(0.06)	(0.06)	(0.06)	(0.06)	(0.06)	(0.06)	(0.07)
Socio-demographics							
Black		− 0.03					0.01
		(0.06)					(0.06)
Other		− 0.03					− 0.10
		(0.08)					(0.07)
Male		− 0.19^∗∗∗^					− 0.14^∗∗∗^
		(0.04)					(0.04)
Florida		− 0.12^∗∗^					− 0.14^∗∗∗^
		(0.05)					(0.05)
New York		− 0.05					− 0.02
		(0.05)					(0.04)
Texas		− 0.15^∗∗∗^					− 0.13^∗∗∗^
		(0.05)					(0.05)
56 or older		0.08^∗∗^					0.06
		(0.04)					(0.04)
Health							
Diabetes			0.02				0.06
			(0.05)				(0.05)
High blood pressure			0.00				− 0.02
			(0.04)				(0.04)
Heart disease			− 0.22^∗∗^				− 0.19^∗∗^
			(0.10)				(0.09)
Asthma			0.02				− 0.00
			(0.05)				(0.05)
Allergies			0.05				0.02
			(0.04)				(0.04)
Other condition			0.09				0.03
			(0.06)				(0.06)
Housing							
Semi-urban/residential				0.04			0.01
				(0.04)			(0.04)
Countryside				− 0.00			− 0.05
				(0.06)			(0.06)
No access to open air				− 0.16^∗∗∗^			− 0.09^∗^
				(0.06)			(0.05)
Work arrangements-losses							
Stopped working					0.16^∗∗∗^		0.07
					(0.06)		(0.06)
Began tele-working					0.15^∗∗^		0.11^∗^
					(0.06)		(0.06)
Not observed working					0.24^∗∗∗^		0.12^∗∗^
					(0.06)		(0.06)
Other					0.20^∗∗^		0.08
					(0.09)		(0.08)
Flag extreme lost income					0.04		0.08
					(0.06)		(0.05)
Lost HH Inc./$1000 Orig					− 0.00		− 0.00
					(0.00)		(0.00)
Lost HH Inc./$1000 Adj.					0.04^∗∗∗^		0.03^∗∗^
					(0.01)		(0.01)
Beliefs-perceptions							
Slightly effective						0.12	0.07
						(0.13)	(0.15)
Moderately effective						0.08	0.03
						(0.12)	(0.14)
Very effective						0.29^∗∗^	0.24^∗^
						(0.12)	(0.14)
Extremely effective						0.36^∗∗∗^	0.27^∗^
						(0.12)	(0.14)
Infected in area						− 0.24^∗∗∗^	− 0.21^∗∗∗^
						(0.07)	(0.08)
More free time						− 0.00	0.01
						(0.03)	(0.03)
More family time						0.07^∗∗^	0.10^∗∗∗^
						(0.03)	(0.04)
Less pollution						0.16^∗∗∗^	0.14^∗∗∗^
						(0.04)	(0.04)
Less noise						0.04	0.03
						(0.04)	(0.04)
Other						0.24^∗∗∗^	0.20^∗∗^
						(0.09)	(0.09)
Observations	781	781	781	781	781	781	781
*R* ^2^	0.009	0.067	0.020	0.022	0.053	0.132	0.202

This table presents estimates from regressions of whether a increased social distancing behaviors after the pandemic started and various individual characteristics. Column (1) only considers income. Column (2) adds socio-demographics to income. Column (3) adds pre-existing health conditions to income. Column (4) adds housing characteristics to income. Column (5) adds work arrangement and losses to income. Column (6) adds beliefs and perceptions about the pandemic to income. Column (7) includes all of these controls in a single specification

Heteroskedastic robust standard errors in parentheses

^∗^*p* < 0.1, ^∗∗^*p* < 0.05, ^∗∗∗^*p* < 0.01

**Table 9:** Factors associated with increased washing hands-wearing mask

	(1)	(2)	(3)	(4)	(5)	(6)	(7)
Income							
Second quintile	0.07	0.07	0.06	0.05	0.07	0.06	0.05
	(0.05)	(0.05)	(0.05)	(0.05)	(0.05)	(0.05)	(0.05)
Third quintile	0.19^∗∗∗^	0.21^∗∗∗^	0.19^∗∗∗^	0.17^∗∗∗^	0.20^∗∗∗^	0.17^∗∗∗^	0.17^∗∗∗^
	(0.05)	(0.05)	(0.05)	(0.05)	(0.05)	(0.05)	(0.05)
Fourth quintile	0.13^∗∗∗^	0.15^∗∗∗^	0.13^∗∗^	0.10^∗^	0.15^∗∗∗^	0.13^∗∗∗^	0.11^∗∗^
	(0.05)	(0.05)	(0.05)	(0.05)	(0.05)	(0.05)	(0.05)
Fifth quintile	0.20^∗∗∗^	0.25^∗∗∗^	0.22^∗∗∗^	0.17^∗∗∗^	0.24^∗∗∗^	0.20^∗∗∗^	0.19^∗∗∗^
	(0.05)	(0.05)	(0.05)	(0.05)	(0.05)	(0.05)	(0.06)
Socio-demographics							
Black		0.09^∗^					0.13^∗∗∗^
		(0.05)					(0.05)
Other		0.08					0.06
		(0.07)					(0.07)
Male		− 0.06^∗^					− 0.01
		(0.03)					(0.03)
Florida		0.02					− 0.00
		(0.04)					(0.04)
New York		0.04					0.05
		(0.04)					(0.04)
Texas		− 0.03					− 0.03
		(0.04)					(0.04)
56 or older		0.14^∗∗∗^					0.13^∗∗∗^
		(0.03)					(0.04)
Health							
Diabetes			− 0.07^∗^				− 0.06
			(0.04)				(0.04)
High blood pressure			0.09^∗∗∗^				0.04
			(0.03)				(0.03)
Heart disease			− 0.05				− 0.05
			(0.08)				(0.07)
Asthma			0.05				0.03
			(0.04)				(0.04)
Allergies			0.08^∗∗^				0.08^∗∗^
			(0.03)				(0.03)
Other condition			0.08^∗^				0.05
			(0.05)				(0.04)
Housing							
Semi-urban/residential				− 0.02			− 0.06^∗^
				(0.03)			(0.03)
Countryside				− 0.04			− 0.07
				(0.05)			(0.05)
No access to open air				− 0.12^∗∗^			− 0.07
				(0.05)			(0.05)
Work arrangements-losses							
Stopped working					0.19^∗∗∗^		0.13^∗∗^
					(0.05)		(0.05)
Began tele-working					0.13^∗∗^		0.13^∗∗^
					(0.06)		(0.05)
Not observed working					0.19^∗∗∗^		0.08
					(0.05)		(0.05)
Other					0.24^∗∗∗^		0.20^∗∗∗^
					(0.07)		(0.06)
Flag extreme lost income					− 0.02		− 0.03
					(0.05)		(0.05)
Lost HH Inc./$1000 Orig					− 0.00^∗∗^		− 0.00
					(0.00)		(0.00)
Lost HH Inc./$1000 Adj.					0.02^∗∗^		0.02^∗∗^
					(0.01)		(0.01)
Beliefs-perceptions							
Slightly effective						− 0.03	− 0.04
						(0.13)	(0.13)
Moderately effective						0.10	0.08
						(0.12)	(0.12)
Very effective						0.22^∗^	0.19
						(0.11)	(0.12)
Extremely effective						0.30^∗∗∗^	0.24^∗∗^
						(0.11)	(0.12)
Infected in area						− 0.20^∗∗∗^	− 0.20^∗∗∗^
						(0.07)	(0.07)
More free time						− 0.04	− 0.04
						(0.03)	(0.03)
More family time						− 0.02	0.00
						(0.03)	(0.03)
Less pollution						0.09^∗∗∗^	0.09^∗∗∗^
						(0.03)	(0.03)
Less noise						0.05	0.04
						(0.03)	(0.03)
Other						0.09	0.05
						(0.07)	(0.07)
Observations	891	891	891	891	891	891	891
*R* ^2^	0.026	0.058	0.046	0.034	0.059	0.120	0.175

This table presents estimates from regressions of whether a increased hand washing or mask wearing behaviors after the pandemic started and various individual characteristics. Column (1) only considers income. Column (2) adds socio-demographics to income. Column (3) adds pre-existing health conditions to income. Column (4) adds housing characteristics to income. Column (5) adds work arrangement and losses to income. Column (6) adds beliefs and perceptions about the pandemic to income. Column (7) includes all of these controls in a single specification

Heteroskedastic robust standard errors in parentheses

^∗^*p* < 0.1, ^∗∗^*p* < 0.05, ^∗∗∗^*p* < 0.01

**Table 10:** Selection of additional variables considered in the analysis

Category	Variable
Housing	
	Current home type
	Current living arrangement
	Elderly exposure at home
	Combinations
Work arrangements	
	Labor status
	Currently working
	Able to work from home
	Ability to work from home (scale)
	Combinations
Losses	
	Work change due to pandemic
	Expected labor income losses
	Expected household income losses
	Combinations
Beliefs-perceptions	
	Belief local infection rate
	Belief Hhspitalization
	Extreme belief indicators
	Combinations

This table lists some of the additional variables that were considered as part of our linear probability model analysis

**Table 11:** Benefits of pandemic by income

	More family time			Less pollution		
Income quintile	Not selected	Selected	Total	Not selected	Selected	Total
First	0.64	0.36	168	0.64	0.36	168
Second	0.67	0.33	190	0.61	0.39	190
Third	0.59	0.41	210	0.56	0.44	210
Fourth	0.53	0.47	243	0.59	0.41	243
Fifth	0.43	0.57	196	0.61	0.39	196

This table summarizes the share of respondents within each income quintile that identified more family time and less pollution as benefits of the pandemic for the US sample

## Footnotes

1 Future work could explore the degree to which the socioeconomic patterns we present here extend to other countries. Findings in Adams-Prassl et al. (2020a) and Briscese et al. (2020) suggest that similar patterns can be seen in the UK and Italy, though Germany might be different. Related, and using the same data set as the one used in this paper, Belot et al. (2020a) examine demographic patterns (focusing on age) in self-protective behaviors across countries.

2 It is worth noting that examining all of the factors could lead to concerns of multiple hypothesis testing. Hence, we limited our analysis a priori to the three behaviors stated above. While they capture different actions, we are in effect testing one hypothesis—whether socioeconomic variables predict self-protective behavior change—using three measures of the outcome variable.

3 Quintile means come from the https://www.taxpolicycenter.org/statistics/household-income-quintiles, administered by the Urban Institute and Brookings Institution.

4 Also related, Borjas (2020) illustrates socioeconomic discrepancies in access to testing in New York City, showing that those without access also tend to be people with the highest probability of a positive test.

5 Wozniak (2020) also provides evidence of declines in well-being due to the pandemic, which relates to Adams-Prassl et al. (2020b), who document a decline in female mental health in the USA due to the lockdown measures to contain the pandemic, that are not explained by other factors such as financial or childcare stress.

6 Cutler et al. (2011) provide an excellent summary and overview of the socioeconomic status-health gradient literature.

7 Papageorge (2016) and Chan et al. (2016) are two studies which examine the history of the HIV epidemic to make broader points about health. More generally, Cawley and Ruhm (2011) provide a summary of the literature that examines cases where individuals make a trade off between controllable behaviors and health outcomes.

8 Many of these ideas were explored in a recent blog post by DeLuca et al. (2020). The current study builds on this piece, in part by using data to test hypotheses it put forth.

9 As a robustness check, we use information based on a respondent’s occupation as a proxy measure for educational attainment and find that main results are unaffected.

10 Some respondents reported very large income losses. Given the difficultly in determining whether these are actual losses or survey errors, we use a dummy variable to flag these values. There are 173 of these observations. Our results are robust to the inclusion of these observations. The only difference is the magnitude on the lost income coefficient. The number in the table reflects the average of the remaining values. Conditional on having lost household income, the average loss was just above $2100.

11 If we include these observations in the “Other” category, our results remain unchanged.

12 In some specifications, we experiment with dropping extreme values under the assumption that they reflect respondent confusion. However, doing so does not alter our results.

13 One might be concerned that self-reported behaviors do not align with actual behaviors and that this bias relates systematically to income. This would be especially concerning if interviews were conducted in-person, but the survey was done on a computer. Moreover, studies specific to Covid-19 that have relied on self-reported information and data collected from devices such as cellphones have corroborated one another (Giuntella et al. 2020; Huckins et al. 2020).

14 To fix ideas, suppose an individual answered 3 (sometimes) for hand washing and 3 (sometimes) for taking public transport before the pandemic and 5 and 4, respectively, during the pandemic. The increase in hand washing accounts for 2 increases in self-protective behaviors and the increase in taking public transportation is recorded as a 1 increment *decrease* in self-protective behaviors. Taken together this results in a net effect of 2 − 1 = 1.

15 We kindly thank a referee for pointing out this concern.

16 This is one way to address pre-pandemic activity levels. We explored others, including different behavior change thresholds and restricting the sample to not include the lowest or highest income quintiles. Generally, our findings were similar under these approaches.

17 We also ran a multinomial logit model where the outcome variable is our work status variable to study which factors predict which types of work changes and find that these patterns hold. These results are available in the Supplemental Appendix of Papageorge et al. (2020).

18 We measure a respondent’s belief in social distancing as the average of their beliefs about the effectiveness of shutting down non-essential businesses, limiting mobility outside the home, and forbidding mass gatherings.

19 Indeed, we must leave open the possibility that, due to our lack of knowledge about the illness, high answers are correct. As of November 2020, this scenario seems increasingly unlikely given yet another surge in cases and hospitalizations in the USA following well-publicized outbreaks in April and July (Madrigal and Kissane 2020).

20 We examined other characteristic associations beyond what is presented in the paper. Table 10 in the Appendix lists some of the other associations we explored.

21 A data set that over-samples non-white individuals would potentially reveal other differences by race or ethnicity in the likelihood of engaging in self-protective behaviors.

22 These estimates can be converted into moments perhaps usable in an epidemiological model. For example, across income quintiles, the probability that an average respondent would increase social distancing behavior if they began tele-working ranged from about 50 to 66%.

23 Similar figures using the lpoly smoother are available in the Supplemental Appendix of Papageorge et al. (2020).

24 According to this variable, 30% of the sample is college educated and the average income is 3.88 for the college educated and 2.78 for people without a college education.

## References

[CR1] Adams-Prassl A, Boneva T, Golin M, Rauh C (2020) Inequality in the impact of the coronavirus shock: evidence from real time surveys. Cambridge-INET

[CR2] Adams-Prassl A, Boneva T, Golin M, Rauh C (2020) The impact of the coronavirus lockdown on mental health: evidence from the US. Working Paper 2020-030. Human Capital and Economic Opportunity Working Group

[CR3] Adda J (2016) Economic activity and the spread of viral diseases: evidence from high frequency data, vol 131, pp 891–941

[CR4] Adolph C, Amano K, Bang-Jensen B, Fullman N, Wilkerson J (2020) Pandemic politics: timing state-level social distancing responses to COVID-19. In: medRxiv 10.1215/03616878-8802162 32955556

[CR5] Akesson J, Ashworth-Hayes S, Hahn R, Metcalfe RD, Rasooly I (2020) Fatalism, beliefs, and behaviors during the COVID-19 pandemic. Working Paper 27245. National Bureau of Economic Research 10.1007/s11166-022-09375-y PMC9161200 35669928

[CR6] Allcott H, Boxell L, Conway J, Ferguson BA, Gentzkow M, Goldman B (2020a) What explains temporal and geographic variation in the early us coronavirus pandemic? Working Paper 27965. National Bureau of Economic Research

[CR7] Allcott H, Boxell L, Conway J, Gentzkow M, Thaler M, Yang DY (2020b) Polarization and public health: partisan differences in social distancing during the coronavirus pandemic. Working Paper 26946. National Bureau of Economic Research 10.1016/j.jpubeco.2020.104254 PMC7409721 32836504

[CR8] Alon TM, Doepke M, Olmstead-Rumsey J, Tertilt M (2020). The impact of COVID-19 on gender equality. Covid Econ.

[CR9] Andersen M (2020) Early evidence on social distancing in response to COVID-19 in the United States. Tech. rep. SSRN

[CR10] Arango T, Cowan J (2020) Gov. Gavin Newsom of California Orders Californians to Stay at Home. https://www.nytimes.com/2020/03/19/us/California-stay-at-home-order-virus.html (visited on 10/20/2020)

[CR11] Ashraf BN (2020) Socioeconomic Conditions, Government Interventions and Health Outcomes During COVID-19, vol 37, pp 141–162

[CR12] Barbash Fred, Horton A (2020) Florida Governor Issues Coronavirus Stay-At-Home Order After Heavy Criticism. https://www.washingtonpost.com/nation/2020/04/01/coronavirus-florida-desantis/ (10/20/2020)

[CR13] Barrios JM, Hochberg Y (2020) Risk perception through the lens of politics in the time of the COVID-19 pandemic. Working Paper 27008. National Bureau of Economic Research

[CR14] Barth D, Papageorge NW, Thom K (2020). Genetic endowments and wealth inequality. J Polit Econ.

[CR15] Béland Louis-Philippe, Brodeur A, Wright T (2020) Covid-19, stay-at-home orders and employment: evidence from CPS data. Discussion Paper 13282. IZA

[CR16] Belot M, Choi S, Jamison J, Papageorge NW, Tripodi E, van den Broek-Altenburg E (2020a) Unequal consequences of Covid 19 across age and income: representative evidence from six countries 10.1007/s11150-021-09560-z PMC8025452 33841055

[CR17] Belot M, Choi S, Jamison J, Papageorge NW, Tripodi E, van den Broek-Altenburg E (2020). Six-country survey on Covid-19. Covid Econ.

[CR18] Bonacini L, Gallo G, Patriarca F (2021). Identifying policy challenges of COVID-19 in hardly reliable data and judging the success of lockdown measures. J Popul Econ.

[CR19] Bonacini L, Gallo G, Scicchitano S (2021). Working from home and income inequality: risks of a ‘new normal’ with Covid-19. J Popul Econ.

[CR20] Borjas GJ (2020). Demographic determinants of testing incidence and COVID-19 infections in New York City neighborhoods. Covid Econ.

[CR21] Briscese G, Lacetera N, Macis M, Tonin M (2020) Compliance with Covid-19 social-distancing measures in Italy: the role of expectations and duration. Discussion Paper 13092. IZA 10.1016/j.socec.2023.101983 PMC9870805 36714370

[CR22] Carroll CD, Crawley E, Slacalek J, White MN (2020) Modeling the consumption response to the CARES Act. Working Paper 27876. National Bureau of Economic Research

[CR23] Cawley J, Ruhm CJ (2011) The economics of risky health behaviors. In: Handbook of Health Economics, vol 2. Elsevier, pp 95–199

[CR24] Chan TY, Hamilton BH, Papageorge NW (2016) Health, risky behaviour and the value of medical innovation for infectious disease, vol 83, pp 1465–1510

[CR25] Chiou L, Tucker C (2020) Social distancing, internet access and inequality. Working Paper 26982. National Bureau of Economic Research

[CR26] CMS (2011) At risk: pre-existing conditions could affect 1 in 2 Americans. https://www.cms.gov/CCIIO/Resources/Forms-Reports-and-Other-Resources/preexisting (visited on 10/20/2020)

[CR27] Coibion O, Gorodnichenko Y, Weber M (2020). The cost of the COVID-19 crisis: lockdowns, macroeconomic expectations, and consumer spending. Covid Econ.

[CR28] Cutler DM, Lleras-Muney A, Vogl T (2011). Socioeconomic status and health: dimensions and mechanisms. The Oxford Handbook of Health Economics.

[CR29] De Avila J (2020) Gov. Cuomo orders all workforce in state to stay home. https://www.wsj.com/articles/new-york-gov-cuomo-orders-all-nonessential-workers-in-state-to-stay-home-11584718223 (visited on 10/20/2020)

[CR30] Delavande A, Perry M, Willis RJ (2006) Probabilistic thinking and social security claiming. Working Paper 129. Michigan Retirement Research Center

[CR31] DeLuca S, Papageorge NW, Kalish E (2020) The unequal cost of social distancing. Available at https://coronavirus.jhu.edu/from-our-experts/the-unequal-cost-of-social-distancing

[CR32] Fairlie RW, Couch K, Xu H (2020) The impacts of COVID-19 on minority unemployment: first evidence from April 2020 CPS microdata. Working Paper 27246. National Bureau of Economic Research

[CR33] Ferrer RA, Klein WilliamMP (2015). Risk perceptions and health behavior. Curr Opin Psychol.

[CR34] Giuntella O, Hyde K, Saccardo S, Sadoff S (2020) Lifestyle and mental health disruptions during Covid-19. Discussion Paper 13569. IZA 10.1073/pnas.2016632118 PMC7936339 33571107

[CR35] Goodman R, Schulkin D (2020) Timeline of the coronavirus pandemic and U.S. response. https://www.justsecurity.org/69650/timeline-of-the-coronavirus-pandemic-and-u-s-response/ (visited on 10/20/2020)

[CR36] Higgins-Dunn N, Breuninger K, Mangan D (2020) New York Gov. Andrew Cuomo orders all people to wear face coverings in public. https://www.cnbc.com/2020/04/15/new-york-gov-cuomo-to-order-all-people-to-wear-masks-or-face-coverings-in-public.html (visited on 10/20/2020)

[CR37] Huckins JF, daSilva AW, Wang W, Hedlund E, Rogers C, Nepal SK, Wu J, Obuchi M, Murphy EI, Meyer ML, Wagner DD, Holtzheimer PE, Campbell AT (2020). Mental health and behavior of college students during the early phases of the COVID-19 pandemic: longitudinal smartphone and ecological momentary assessment study. J Med Internet Res.

[CR38] Lemire J (2020) Trump wears mask in public for first time during pandemic. https://apnews.com/article/7651589ac439646e5cf873d021f1f4b6 (visited on 10/20/2020)

[CR39] Lillard LA, Willis RJ (2001) Cognition and wealth: the importance of probabilistic thinking. Working Paper 7. Michigan Retirement Research Center

[CR40] Madrigal AC, Kissane E (2020) The worst day of the pandemic since May. https://www.theatlantic.com/science/archive/2020/11/pandemic-coronavirus-hospitalizations-new-record/617061/ (visited on 11/10/2020)

[CR41] Manski CF (2020) Covid-19 policy must take all impacts into account. Scientific American. Available at https://blogs.scientificamerican.com/observations/covid-19-policy-must-take-all-impacts-into-account/

[CR42] Manski CF, Molinari F (2020) Estimating the COVID-19 infection rate: anatomy of an inference problem. Journal of Econom 10.1016/j.jeconom.2020.04.041 PMC7200382 32377030

[CR43] McCarthy J (2020) 43% of U.S. households report preexisting conditions. https://news.gallup.com/poll/269003/households-report-preexisting-conditions.aspx (visited on 10/20/2020)

[CR44] Milani F (2021). COVID-19 outbreak, social response, and early economic effects: a global VAR analysis of cross-country interdependencies. J Popul Econ.

[CR45] Mongey S, Pilossoph L, Weinberg A (2020). Which workers bear the burden of social distancing policies?. Covid Econ.

[CR46] Okubo T (2020). Spread of COVID-19 and telework: evidence from Japan. Covid Econ.

[CR47] Painter M, Qiu T (2020). Political beliefs affect compliance with COVID-19 social distancing orders. Covid Econ.

[CR48] Pampel FC, Krueger PM, Denney JT (2010). Socioeconomic disparities in health behaviors. Ann Rev Sociol.

[CR49] Papageorge NW (2016). Why medical innovation is valuable: health, human capital, and the labor market. Quant Econ.

[CR50] Papageorge NW, Zahn MV, Belot M, van den Broek-Altenburg E, Choi S, Jamison JC, Tripodi E (2020) Socio-demographic factors associated with self-protecting behavior during the Covid-19 pandemic. Working Paper 27378. National Bureau of Economic Research 10.1007/s00148-020-00818-x PMC7807230 33462529

[CR51] Posner RA, Tomas J. P. (1993). Private choices and public health: the aids epidemic in an economic perspective.

[CR52] Qiu Y, Chen X, Shi W (2020). Impacts of social and economic factors on the transmission of coronavirus disease 2019 (COVID-19) in China. J Popul Econ.

[CR53] Rahman AS (2020). Why can’t everybody work remotely? Blame the robots. Covid Econ.

[CR54] Romo V (2020) California Gov. Newsom makes face masks mandatory amid rising coronavirus cases. https://www.npr.org/sections/coronavirus-live-updates/2020/06/18/880583357/california-gov-newsom-makes-face-masks-mandatory-amid-rising-coronavirus-cases (visited on 10/20/2020)

[CR55] Saltiel F (2020). Who can work from home in developing countries. Covid Econ.

[CR56] Shapiro J, Wu S (2011). Fatalism and savings. J Socio-Econ.

[CR57] Siemaszko C (2020) Texas governor mandates mask-wearing across most of state as coronavirus cases surge. https://www.nbcnews.com/news/us-news/texas-governor-mandates-mask-wearing-across-most-state-coronavirus-cases-n1232845 (visited on 10/20/2020)

[CR58] Simonov A, Sacher SK, Dubé J-PH, Biswas S (2020) the persuasive effect of fox news: non-compliance with social distancing during the Covid-19 pandemic. Working Paper 27237. National Bureau of Economic Research

[CR59] Smith AC, Thomas E, Snoswell CL, Haydon H, Mehrotra A, Clemensen Jane, Caffery LJ (2020). Telehealth for global emergencies: implications for coronavirus disease 2019 (Covid-19). J Telemed Telecare.

[CR60] Svitek P (2020a) Gov. Greg Abbott closes bars, restaurants and schools as he anticipates tens of thousands could test positive for coronavirus. https://www.texastribune.org/2020/03/19/texas-restaurants-bars-closed-greg-abbott/ (visited on 10/20/2020)

[CR61] Svitek P (2020b) Gov. Greg Abbott resists calls for statewide shelter-in-place; moves to expand hospital capacity. https://www.texastribune.org/2020/03/22/texas-shelter-in-place-coronavirus-cases/ (visited on 10/20/2020)

[CR62] Svitek P (2020c) Gov. Greg Abbott tells texans to stay home except for essential activity in april. https://www.texastribune.org/2020/03/31/greg-abbott-texas-executive-order-closures/ (visited on 10/20/2020)

[CR63] Viner RM, Russell SJ, Croker H, Packer J, Ward J, Stansfield C, Mytton O, Bonell C, Booy R (2020) School closure and management practices during coronavirus outbreaks including COVID-19: a rapid systematic review. Lancet Child Adolesc Health 10.1016/S2352-4642(20)30095-X PMC7270629 32272089

[CR64] WHO (2020) Pandemic fatigue - reinvigorating the public to prevent Covid-19. Tech. rep. WHO Regional Office for Europe

[CR65] Wozniak A (2020) Disparities and mitigation behavior during COVID-19. Institute Working Paper 32. Opportunity and Inclusive Inclusive Growth Institute: Federal Reserve Bank of Minneapolis

[CR66] Wright AL, Sonin K, Driscoll J, Wilson J (2020) Poverty and economic dislocation reduce compliance with COVID-19 shelter-in-place protocols. Working Paper 2020-40. University of Chicago, Becker Friedman Institute for Economics 10.1016/j.jebo.2020.10.008 PMC7568053 33100443

[CR67] Yancy CW (2020). COVID-19 and african americans. J Am Med Assoc.

[CR68] Zimmermann KF, Karabulut G, Bilgin MH, Doker AC (2020). Inter-country distancing, globalisation and the coronavirus pandemic. World Econ.

